# Evaluating Monitoring Strategies to Detect Precipitation-Induced Microbial Contamination Events in Karstic Springs Used for Drinking Water

**DOI:** 10.3389/fmicb.2017.02229

**Published:** 2017-11-22

**Authors:** Michael D. Besmer, Frederik Hammes, Jürg A. Sigrist, Christoph Ort

**Affiliations:** ^1^Department of Environmental Microbiology, Eawag, Swiss Federal Institute of Aquatic Science and Technology, Dübendorf, Switzerland; ^2^Department of Environmental Systems Science, Institute of Biogeochemistry and Pollutant Dynamics, ETH Zürich, Zurich, Switzerland; ^3^Department of Urban Water Management, Eawag, Swiss Federal Institute of Aquatic Science and Technology, Dübendorf, Switzerland

**Keywords:** water quality monitoring, sampling, microbial dynamics, drinking water, spring water, early warning systems, risk assessment

## Abstract

Monitoring of microbial drinking water quality is a key component for ensuring safety and understanding risk, but conventional monitoring strategies are typically based on low sampling frequencies (e.g., quarterly or monthly). This is of concern because many drinking water sources, such as karstic springs are often subject to changes in bacterial concentrations on much shorter time scales (e.g., hours to days), for example after precipitation events. Microbial contamination events are crucial from a risk assessment perspective and should therefore be targeted by monitoring strategies to establish both the frequency of their occurrence and the magnitude of bacterial peak concentrations. In this study we used monitoring data from two specific karstic springs. We assessed the performance of conventional monitoring based on historical records and tested a number of alternative strategies based on a high-resolution data set of bacterial concentrations in spring water collected with online flow cytometry (FCM). We quantified the effect of increasing sampling frequency and found that for the specific case studied, at least bi-weekly sampling would be needed to detect precipitation events with a probability of >90%. We then proposed an optimized monitoring strategy with three targeted samples per event, triggered by precipitation measurements. This approach is more effective and efficient than simply increasing overall sampling frequency. It would enable the water utility to (1) analyze any relevant event and (2) limit median underestimation of peak concentrations to approximately 10%. We conclude with a generalized perspective on sampling optimization and argue that the assessment of short-term dynamics causing microbial peak loads initially requires increased sampling/analysis efforts, but can be optimized subsequently to account for limited resources. This offers water utilities and public health authorities systematic ways to evaluate and optimize their current monitoring strategies.

## Introduction

Adequate monitoring of drinking water quality is one of the key components ensuring that safe and clean drinking water is produced and provided to customers. Short-term microbial dynamics at the scale of minutes to weeks are to be expected in drinking water systems. This can result from natural fluctuations in raw water sources (e.g., precipitation events, snowmelt) as well as operational changes (e.g., filter backwashing, intermittent flow) during treatment (Stevenson, 1997; Pronk et al., 2006; Madrid and Zayas, 2007; Stadler et al., 2008; Bakker et al., 2013). Short-term dynamics and especially peak concentrations strongly influence water quality—and the infection risk in the case of pathogens—especially in raw water but also in treated water (Gauthier et al., 2001; Kistemann et al., 2002; Vreeburg et al., 2004; Farnleitner et al., 2005; Signor and Ashbolt, 2006; Astrom et al., 2007; Pronk et al., 2007; Stadler et al., 2008). Furthermore, many small water utilities using spring water or groundwater have either no or very limited water treatment in place and are thus directly exposed to changes and associated risks in raw water quality. In spite of this, current monitoring practice is often not designed to detect short-term dynamics (Stadler et al., 2008). In fact, it is not uncommon for small utilities to sample on a quarterly or monthly frequency only. This is due to limited financial and logistic resources but also due to the limited existing knowledge on microbial short-term dynamics *per se*.

The general problem of a low sampling frequency is that it represents a system's dynamics insufficiently and especially does not reflect transient changes in water quality. This was considered previously for seasonal changes and water quality violations in river water (Loftis and Ward, 1980; Casey et al., 1983). More recent studies on chemical water quality monitoring in surface waters included optimization strategies for quarterly and monthly sampling (Do et al., 2013; Naddeo et al., 2013; Liu et al., 2014) and illustrations of the large uncertainties remaining even with weekly sampling (Skeffington et al., 2015). Similarly, many dynamics in drinking water production systems occur at short time scales and can thus be easily missed by conventional sampling regimes (i.e., infrequent, manual grab sampling) (Signor and Ashbolt, 2006; Madrid and Zayas, 2007). For example, systems treating surface water tend to be driven by diurnal cycles and thus dynamics have a time scale of hours to days (Besmer et al., 2014). Technical systems that are influenced by human activity can have dynamics of virtually any time scale and different dynamics are often superimposed on each other (Besmer et al., 2016). Many of the dynamics are diurnal or otherwise periodic (i.e., regular) because technical systems include defined, regular operational procedures (e.g., backwashing of filters) and the typical time scale is minutes to hours (Besmer and Hammes, 2016). Arguably, both periodic and even more so aperiodic deviations/peaks in microbial quality can be viewed as time periods of increased contamination risk and hence should be investigated in more detail to verify or exclude contamination. From a practical point of view, it is particularly relevant to know if/when a contamination event occurs and what its magnitude is.

One obvious solution is online monitoring. For drinking water, Janke et al. (2006) showed the advantage of physicochemical online monitoring over conventional monitoring with sampling frequencies below 24 h in the context of deliberate sabotage. Emerging online monitoring tools were further summarized by Storey et al. (2011) and emerging technologies for measuring microbial variables online and at high frequency have been demonstrated in various settings and include flow cytometry (FCM), enzymatic activity, and optical detection (Besmer et al., 2014; Ryzinska-Paier et al., 2014; Hojris et al., 2016). While promising, it is highly unlikely that widespread routine application of microbial online monitoring will be implemented in the near future, due to financial constraints and legal limitations. Therefore, we argue that smarter and more efficient monitoring strategies, based on available and/or affordable equipment, are needed. To optimize monitoring strategies, the drivers and relevant time scales of the dynamic need to be understood (ISO, 2006). To our knowledge, this has not been done adequately for microbial monitoring in spring water, partially due to the lack of high-resolution data sets to date.

The present study focuses on karstic springs, which are used as drinking water sources throughout Europe (Scheidleder, 1999). The porous nature of the karstic geology enables microbial contamination of the spring water with infiltrating surface water after localized precipitation events (Field and Nash, 1997; Farnleitner et al., 2005). We assessed historical records of conventional monitoring data of karstic spring water and compared them with newly collected high-frequency data sets. The purpose was to systematically assess the temporal variability of spring water microbial quality, and to evaluate suitable monitoring strategies to accurately capture those dynamics. The specific goals of this study were: (1) to assess limitations of the current monitoring practice of regular but infrequent grab sampling for microbial water quality control; (2) to illustrate the effect of sampling frequency on the probabilities of detecting precipitation-induced microbial events in karstic spring water; and (3) to suggest a targeted sampling strategy for microbial water quality changes in karstic spring water after precipitation events. The novelty of this study is the investigation of the effect of different monitoring strategies on the information gained from sampling, based on systematic analysis of temporally highly resolved measurements of bacterial concentrations.

## Materials and methods

### Study sites, samples, and data sets

Data was collected from two springs (A and B) in a karstic region in the Northeast of Switzerland. The focus was on raw spring water prior to any treatment. An overview of the experimental work and data sets is given in Table 1. Auto-sampler campaigns and subsequent detection with manual FCM and plating for both heterotrophic plate count (HPC) and indicator organisms were carried out for this study specifically in spring A, during two subsequent weeks. Within this period, two dry-weather periods were sampled every hour for 24 h each. In addition, a 48-h sampling campaign was carried out with samples taken every hour on two consecutive days after a precipitation event. An online FCM data set was generated for spring B, of which a subset was published in Besmer and Hammes (2016). Here, the full 99-day data set is used and the focus is on systematic analysis. In addition, two long-term data sets (2002–2015) of conventional monitoring based on infrequent (i.e. quarterly/monthly) grab sampling and cultivation-based detection methods were provided by the Food Safety and Veterinary Office Basel-Landschaft (FSVO BL) for springs A and B. Precipitation data in parallel to the intensive microbial measurements (2014–2015) was available from a temporary meteorological station located close to the two investigated springs. Additional, long-term precipitation data (2002–2015) was obtained from the Swiss Federal Office of Meteorology and Climatology (MeteoSwiss) for the permanent meteorological station closest to the study region. Spring discharge measurements were provided by the water utilities.

**Table 1:** Overview table of sampling campaigns and data sets used for the different analyses in this study.

**Data sets**	**Spring A**		**Spring B**	
Auto-sampler campaign Total cell concentration Heterotrophic plate count Indicator organisms	December 2014 3 × 24 h every 1 h	Figure 1	June 2015 1 × 48 h every 1 h	Figure S1
Local precipitation	2014/2015 2 years every 30 min	Figure 1	2014/2015 2 years every 30 min	Figure 2, Figures S1–S3
Spring discharge	Aug 2014–Jul 2015 1 year every 30 min	Figure 1	March–July 2015 99 days every 30 min	Figure S1
Online flow cytometry Total cell concentration	–	–	March–July 2015 99 days every 15 min	Figure 2, Figures S1–S3
Conventional monitoring Heterotrophic plate count Indicator organisms	2002–2015 14 years quarterly/monthly	Figure 1, Table 2	2002–2015 14 years quarterly/monthly	Figure S1
Regional precipitation	2002–2015 14 years every 10 min	in text	2002–2015 14 years every 10 min	in text

### Sampling

Grab samples were taken according to the standard procedures of the FSVO BL, which is an accredited state agency for inspection in accordance with standard ISO 17020:2012 (ISO, 2012) as well as an accredited testing laboratory in accordance with standard ISO 17025:2015 (ISO, 2005). In short, water samples were collected from disinfected (flame treatment of taps prior to sampling), flowing taps or directly from the spring outflow. A portable and programmable auto-sampler (ISCO 6712, Teledyne ISCO Inc., Lincoln, USA) was used for automated sampling. Samples (800 ml) were drawn every hour into sterilized plastic bottles [rinsed thoroughly with hypochlorite solution (1% active chlorine) and 3 times with nanopure water (deionized, 0.22 μm filtered) water before pasteurization at 60°C for 1 h]. The sampling tube was automatically rinsed and purged three times before each sample to avoid stagnation and cross contamination. All samples were transported and stored at 4°C and processed within 24 h.

### Manual detection methods

Heterotrophic plate count (HPC) plating was done in accordance with the standard ISO 4833:2003 (ISO, 2003) spread plating method by an accredited laboratory. In short, 1 ml of a water sample was evenly distributed on an agar plate and then incubated for 72 h at 30°C. The number of formed colonies was subsequently counted. For indicator organism plating, the standard 9308-1:2000 (ISO, 2000) and 1406.1 (SLMB, 2007) membrane filtration and plating methods for the enumeration of *Escherichia coli* (*E. coli*) and Enterococcus respectively were used. In short, 100 ml of a water sample were filtered through a 0.45 μm filter, which was then placed on an agar plate and incubated for 24 h at 37°C. The number of formed colonies was subsequently counted.

Manual FCM measurements of total cell concentration (TCC) were done based on the reference method 333.1 (SLMB, 2012). In short, 500 μl of the water samples were pre-warmed for 3 min at 37°C and then stained with the fluorescent stain SYBR Green I (Life Technologies, Eugene OR, USA; final concentration 1:10,000). After 10 min of incubation at 37°C in the dark, 100 μl of a sample were measured on an Accuri C6 flow cytometer (BD Accuri, San Jose CA, USA) at a flow rate of 66 μl min^−1^ with a lower threshold on the green fluorescence (FL1-H) channel of 1,000. Fixed gates were applied in the Accuri C6 CFlow software to separate bacteria from background signals (Prest et al., 2013).

### Automated detection method: online flow cytometry

For online FCM, water was sampled directly from a bypass with continuous flow by an automated sampling, staining, and incubation module connected to an Accuri C6 flow cytometer (BD Accuri, San Jose CA, USA) as described previously (Besmer et al., 2014). In short, water samples were drawn discretely every 15 min and mixed with a fluorescent stain [SYBR Green I (Life Technologies, Eugene OR, USA); final concentration 1:10,000]. This mixture was incubated for 10 min at 37°C before transfer to the flow cytometer for measurement at a flow rate of 66 μl min^−1^ for 90 s with a lower threshold on the green fluorescence (FL1-H) channel of 1,000. After each sampling and measurement cycle, the staining module was rinsed with nanopure water (deionized, 0.22 μm filtered). In addition, an extended cleaning cycle with hypochlorite and detergent was performed after every 100 samples. For data analysis, files were exported for batch processing with custom software. Fixed gates were applied to separate bacteria from background signals (Prest et al., 2013).

### Systematic analysis of monitoring strategies

#### Event definition

A preliminary analysis of high resolution TCC and precipitation data in spring B indicated substantial TCC increases after precipitation events with total volumes exceeding 10 mm within 24 h. Due to the time scale of the system response (i.e., TCC increase/decrease after precipitation), we added a second criterion that no new precipitation event should start within 48 h.

#### Evaluation of different monitoring strategies

We tested three different monitoring strategies to assess their efficacy in *TCC event detection* and *TCC peak concentration estimation*: (1) sampling at pre-defined, constant time intervals, (2) random grab samples taken during working hours only (Skeffington et al., 2015), and (3) targeted sampling (triggered by precipitation events). The analysis was performed by subsampling the high-resolution TCC data set, which was assumed to represent the “true” temporal evolution of bacterial concentration. Based on the definition of relevant precipitation events above, the TCC data set was divided into separate TCC events to be evaluated. Then, strategies 1 and 2 were tested for five different sampling frequencies over the entire 99-day monitoring period to detect these defined TCC events (with different total numbers of samples): quarterly (1 sample), monthly (3 samples), weekly (14 samples), bi-weekly (28 samples) and daily [99 (all days, strategy 1) and 70 (all working days, strategy 2)]. For strategy 1, the maximum number of possible realizations (resulting from sampling interval and TCC event duration) was evaluated. For strategy 2, 10,000 random realizations were evaluated. For strategy 3, three (sub-)samples were taken, 24, 48, and 72 h after the criterion for the precipitation volume was met.

#### Statistical analysis

Two criteria were assessed for the evaluation of the different monitoring strategies for each of the 11 events defined above: (1) The efficacy in *detecting a TCC event*. This was quantified for each event by the probability of taking a sample during an event. (2) The *accuracy in estimating the TCC peak concentration*. This was quantified for each event by the ratio (R) of the sampled maximum divided by the true maximum. Subsequently, for the comparison of monitoring strategies the 25%, 50% (median), and 75% quartiles were used. The three quartiles of all realizations were calculated for each individual event for each sampling frequency and both sampling strategies 1 and 2. In the case of the targeted monitoring strategy (3), the second step was performed with the highest measurement (i.e., closest to the true maximum) of the three samples per individual event. The second step was additionally performed excluding events 7 and 8, which showed no substantial/relevant TCC increase despite fulfilling the sampling trigger criterion (10 mm within 24 h).

#### Software

All data analysis was carried out in R (R Development Core Team, 2008) using standard packages (the full code is available in the Supplementary Information).

## Results and discussion

The overall goal of this study was to systematically assess the temporal variability of karstic spring water microbial quality and suitable monitoring strategies to accurately capture the prevalent dynamics. To this end, we investigated two karstic springs from the same geographical area based on the availability of a large historical data set (Spring A) and the opportunity to install new online monitoring equipment (Spring B). The investigations in Spring A (section *Precipitation-Induced Dynamics and Current Grab Sampling Practice*) cover the effect of precipitation events and the implications resulting from infrequent grab sampling practices by (1) illustrating the link between precipitation events, increased spring discharge, and microbial contamination, (2) establishing the suitability of flow cytometric TCC as a useful parameter to follow bacterial dynamics in these springs, and (3) estimating how many precipitation-induced contamination events are missed by conventional monitoring. A detailed analysis of online FCM data from Spring B (section *Increased Sampling Frequency Improves Contamination Event Detection*) illustrates how increasing the sampling frequency increases the probability of detecting microbial contamination events. From this data, we argue for an optimized, targeted monitoring strategy with event-based triggering and appropriate sampling intervals (section *Optimizing Contamination Event Detection Through Targeted Sampling*).

### Precipitation-induced dynamics and current grab sampling practice (Spring A)

**Figure 1:**
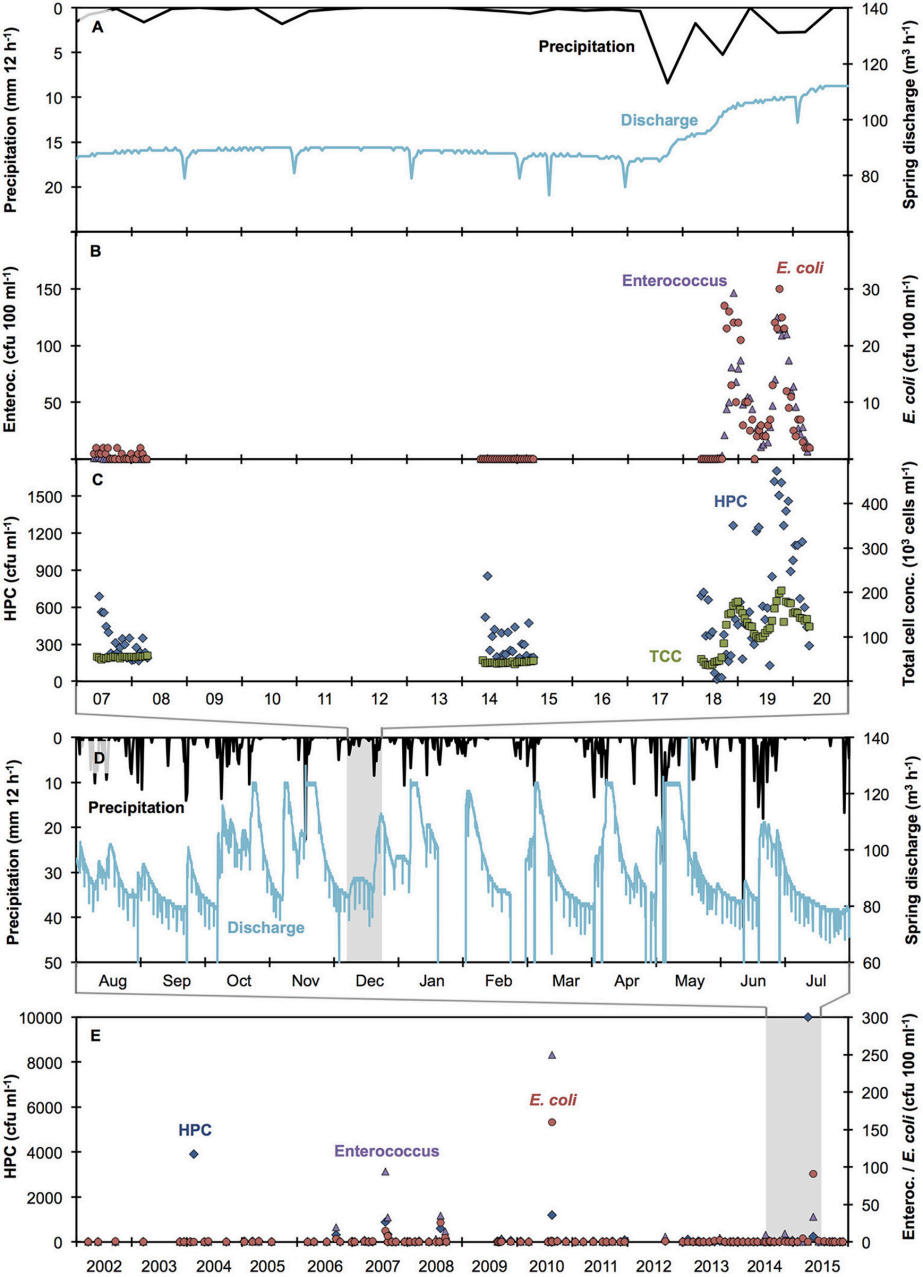
Evaluation of raw spring water quality (Spring A) over different time scales: **(A,D)** precipitation and spring discharge measurements [hourly measurements; 2 weeks **(A)** and one year **(D)** respectdively], **(B,C)** auto-sampler measurements analyzed with conventional plating methods for the indicator organisms Enterococcus (purple triangles), *E. coli* (red circles), and HPC (blue diamonds) as well as flow cytometric total cell concentration (green squares), **(E)** conventional grab sampling (quarterly to monthly; 14 years; *n* = 100) analyzed with conventional plating methods for the indicator organisms Enterococcus (purple triangles), *E. coli* (red circles), and HPC (blue diamonds). Short-term drops in spring discharge are due to water being discarded for operational reasons. Maximum spring discharge was 125 m^3^ h^−1^ for operational reasons (excessive water was discarded).

Time-resolved data from Spring A shows that localized precipitation in excess of 10 mm in 24 h causes increased discharge and microbial contamination of karstic spring water (Figures 1A–C), in agreement with previous studies (Stadler et al., 2008; Goldscheider et al., 2010; Butscher et al., 2011; Page et al., 2017) and analogous measurement campaigns in other springs in this region and at other times of the year (Figure S1; other data not shown). As such, multiple precipitation events will result in multiple contamination events, characterized by both the frequency and magnitude of increases in relevant microbial variables. During dry-weather periods (Figure 1A), low concentrations of indicator organisms (0–2 cfu 100 ml^−1^) were detected (Figure 1B), suggesting a minor input from precipitation-independent sources. In contrast, the 48-h sampling after a localized precipitation event revealed two distinct peaks in both Enterococcus and *E. coli* concentrations (up to 150 and 30 cfu 100 ml^−1^ respectively, Figure 1B). Time series of both indicator organisms followed a clear trend, with rapid increases and slightly slower decreases after peaking (Figure 1B). HPC exceeded 1,700 cfu ml^−1^ after precipitation events and were lower (314.5 ± 149.7 cfu ml^−1^) during the dry weather periods (Figure 1C). Compared to results for indicator organisms (above), HPC results were more variable between consecutive time points, making the contamination event difficult to track. TCC was low (48,600 ± 6,400 cells ml^−1^) during dry weather periods and reached more than 200,000 cells ml^−1^ after precipitation events (Figure 1C). Of the four microbial measurements, TCC evolved most consistently (i.e., lowest variation between consecutive time points). From this we draw a first conclusion that TCC data is particularly suitable to describe both dry weather conditions as well as precipitation-induced dynamics in bacterial concentrations in karstic springs. Importantly, the temporal evolution of indicator organisms and TCC was comparable, although no direct proportionality was found (data not shown).

When expanding the observation period to a detailed set of precipitation and discharge data during 12 consecutive months (2014–2015), it is evident that a total of 31 major precipitation events occurred, which exceeded 10 mm in 24 h (Figure 1D). All of these precipitation events caused noticeable increases in spring discharge (Figure 1D). Hence, for this spring and this time period, precipitation events were frequent and thus precipitation-induced contamination events can be expected to be equally frequent. From these combined observations, we infer that historical precipitation data can reasonably be used to estimate the number of contamination events in the spring water.

Based on this argument, we subsequently evaluated regional precipitation data during 14 years (2002–2015) and found that 380 major precipitation events (>10 mm within 24 h) occurred (data not shown). In the same historical period, a total of 100 water samples was analyzed by the responsible authority in the course of routine monitoring campaigns of this spring (quarterly samples from 2002 to 2012 and monthly samples from 2013 to 2015) (Table 2, Figure 1E). Of these conventional grab samples, < 30% tested positive for indicator organisms, Table 2). Based on the historical data, Spring A appears to have experienced rather few contamination events and most of these were of moderate magnitude (Figure 1E). Furthermore, because the number of grab samples with elevated bacterial concentrations was low, it is conceivable that they may be (falsely) considered to be outliers due to contamination during sampling or analysis. In stark contrast, the results from the auto-sampler campaign (Figures 1A–C) strongly suggest that (1) the spring actually experienced substantial bacterial peak loads after precipitation events and (2) the high concentrations of Enterococcus and *E. coli* occasionally detected with grab sampling (Figure 1E) were probably real detections of precipitation-induced contamination events.

**Table 2:** Monitoring data for indicator organisms during 14 years as part of conventional monitoring of drinking water microbial quality by responsible authorities based on infrequent quarterly (Q) (2002–2012) and monthly (M) (2013–2015) grab sampling in Spring A, displayed in Figure 1.

			**Samples** >**0 cfu 100 ml**^**−1**^									
					**Concentration**							
**Detected organisms**	**Number of samples analyzed**		**Number of positives**		**Median**		**Average**		**Std. dev**.		**Maximum**	
	**Q**	**M**	**Q**	**M**	**Q**	**M**	**Q**	**M**	**Q**	**M**	**Q**	**M**
Enterococcus	63	37	18 (29%)^a^	9 (24%)^a^	3.0	3.0	26.3	7.8	60.3	10.5	250.0	34.0
*E. coli*	63	37	14 (22%)^a^	8 (22%)^a^	1.5	2.0	16.3	13.3	42.0	31.4	160.0	91.0
Both	63	37	9 (14%)^a^	4 (11%)^a^	–	–	–	–	–	–	–	–

a *Percentage of samples >0 cfu 100 ml^−1^ in all samples analyzed*.

On the above-discussed premise that the 380 major precipitation events between 2002 and 2015 most likely caused substantial increases in spring discharge and bacterial concentrations, the quarterly sampling strategy (2002–2012) only detected at most 6% (18 measured samples >0 cfu 100 ml^−1^ vs. 292 major precipitation events). When taking into account the observation of occasionally low detection of indicator organisms during dry-weather periods (Figure 1B) and the median values for the samples above 0 cfu 100 ml^−1^ being similarly low (Table 2, Figure 1E), the actual detection of precipitation-induced contamination events was probably even lower. Analogously, the monthly sampling strategy (2013–2015) detected at most 10% of contamination events (9 measured samples >0 cfu 100 ml^−1^ while 88 major precipitation events were recorded).

In summary, the data shows that the conventional monitoring strategy based on infrequent grab sampling was ineffective in detecting the frequency of precipitation-induced contamination events in karstic springs and failed to quantify the magnitude of these events. Importantly, these findings were not limited to this specific spring (Spring A) and were confirmed in a similar assessment of Spring B, with a known record of generally high microbial loads (Figure S1).

### Increased sampling frequency improves contamination event detection (Spring B)

**Figure 2:**
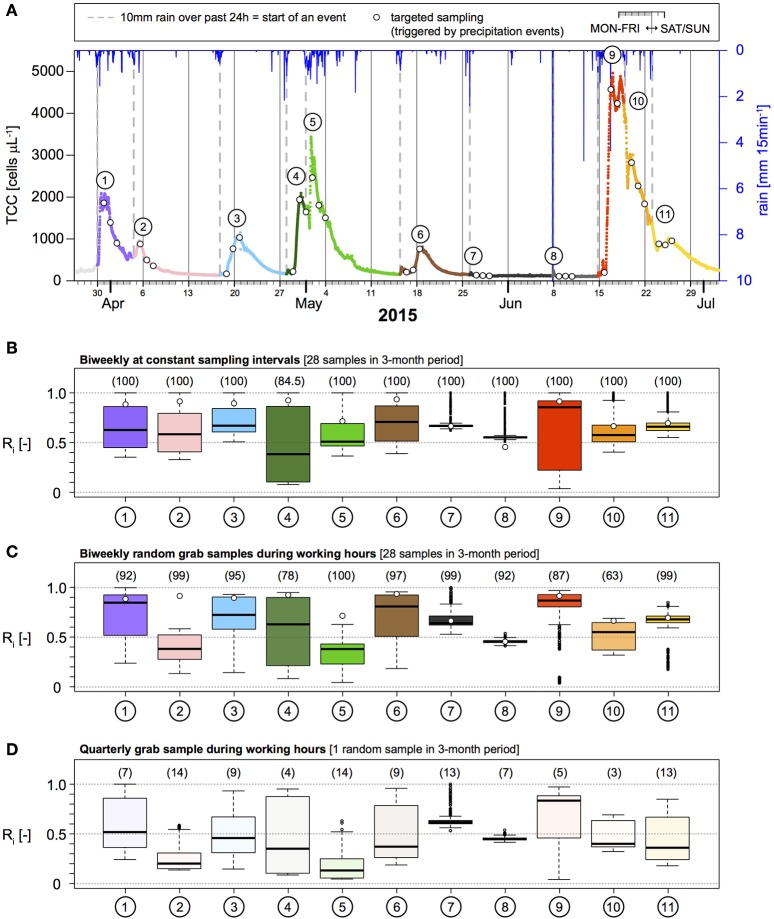
Overview of the 3-month observation period for total cell concentration (TCC) and precipitation **(A)**. A minimum of 10 mm of rainfall recorded over 24 h marks the start of a TCC event (dashed gray lines). Each TCC event is numbered and color-coded throughout the figure. Circles indicate the three samples from the targeted sampling (i.e., 24, 48, and 72 h after the start of a TCC event). **(B–D)** Show the distribution of the accuracy of peak concentrations of TCC for different monitoring strategies based on multiple realizations. Numbers in brackets and opacity of boxes indicate the probability of TCC event detection. White circles indicate the best result of the targeted sampling for direct comparison. Boxes represent 25%, 50% (i.e., median, black lines), and 75% quartiles. Whiskers represent 1.5-fold interquartile ranges or minima/maxima when outside this range.

An obvious strategy to improve the probability of detecting and correctly quantifying contamination events in any system is to sample more frequently. In this respect, continuous online microbial monitoring presents an interesting future solution (Besmer et al., 2014; Besmer and Hammes, 2016; Page et al., 2017). Online FCM data from Spring B (7,878 measurements at 15 min interval in 3 months) shows the frequency and magnitude of TCC increases during precipitation-induced contamination events (Figure 2A). Based on the precipitation event definition above (>10 mm in 24 h), a total of 11 precipitation events, each followed by an increase in TCC, were identified (Figure 2A). We subsequently performed a theoretical sub-sampling of this online TCC data set to evaluate different monitoring strategies. The probability to detect elevated TCC as a result of precipitation events was assessed for (1) constant sampling intervals and (2) random sampling during working hours, at frequencies of quarterly (1 sample), monthly (3 samples), bi-weekly (28 samples), weekly (14 samples), and daily (99 samples) sampling.

The monitoring strategy with constant sampling intervals performed slightly but consistently better compared to the same number of samples taken randomly during working hours, but the differences were small (Table 2, Figures 2B,C). For the widespread conventional monitoring strategy of quarterly or monthly sampling, the average probability to detect an individual event of elevated TCC was 9.6 and 28.9% respectively at constant sampling intervals. This probability increased to 85.5% for bi-weekly and to 98.6% for weekly sampling and reached 100% for daily sampling (Table 3, Figure 2, Figure S2). If samples were taken randomly but during working hours only, the probabilities to detect the TCC events were consistently lower for the same number of samples but reached 100% for daily sampling as well (Table 3, Figure S3). While daily sampling is effective in detecting the events, it is not a logistically, practically or financially realistic strategy for routine applications. At least bi-weekly sampling was needed to reach detection probabilities >90% for this specific spring (Table 2, Figures 2B,C), which is still a very resource-intensive approach. Nevertheless, we used this sampling frequency as the example for further comparison with optimization strategies.

**Table 3:** Overview of the different monitoring strategies and the resulting (1) probability to detect precipitation-induced TCC events and (2) accuracy of peak concentration estimations of bacteria in karstic spring water during a 3-month observation period (Figure 2, Spring B).

**Monitoring strategy**		**Probability of TCC event detection**			**Estimation of TCC peak concentration (*****R*** = **sampled maximum divided by true maximum)**			
		***n***	**Average**	**Range (%)**	**Median (%)^a^**	**25% Quartile (%)^a^**	**75% Quartile (%)^a^**	**Median range (%)^b^**
Constant interval	Quarterly	1	10	3–16	43 (31)	19 (17)	61 (59)	14–84
	Monthly	3	29	10–48	44 (31)	19 (17)	62 (59)	14–84
	Weekly	14	86	42–100	54 (47)	34 (29)	67 (68)	33–84
	Bi-weekly	28	99	85–100	64 (64)	52 (49)	80 (84)	38–86
	Daily	99	100	–	87 (89)	72 (82)	93 (93)	56–94
Randomly (working hours)	Quarterly	1	9	2–15	41 (32)	20 (17)	62 (60)	16–83
	Monthly	3	24	9–37	43 (36)	21 (18)	63 (64)	16–83
	Weekly	14	71	35–91	51 (50)	32 (26)	68 (74)	23–85
	Bi-weekly	28	91	63–100	61 (62)	41 (38)	84 (86)	38–87
	Daily	70	100	–	81 (87)	55 (58)	92 (92)	46–93
Targeted		33	100	–	89 (90)	69 (72)	92 (92)	46–94

**Figure 3:**
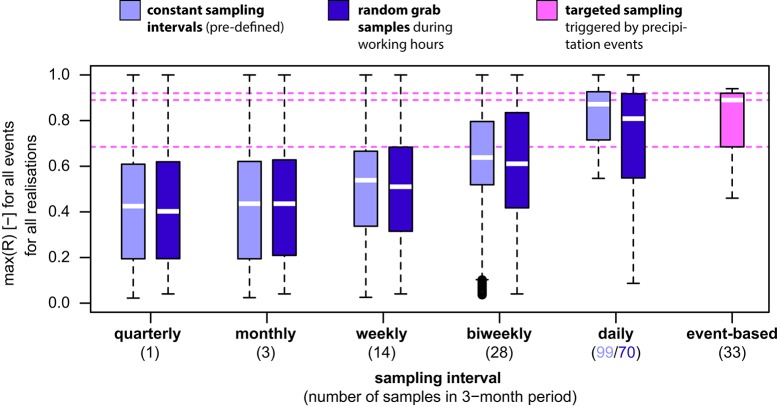
Comparison of estimation of peak concentration (*R* = sampled maximum divided by true maximum) for different monitoring strategies and number of samples calculated for all realizations for all 11 TCC events (see Table 3 and Table S1 for detailed values). For the targeted sampling, the values were calculated for one realization only for all 11 TCC events and the best R values (i.e., closest to the true value) out of three samples taken per TCC event were used. White lines represent the median, boxes represent 25/75% quartiles, whiskers represent 1.5 times interquartile ranges (or minima/maxima). Horizontal dotted lines are 25%, 50% (median), and 75% quartiles of the targeted sampling for comparison with other strategies and numbers of samples.

*For the constant sampling interval and random sampling, multiple possible realizations were statistically summarized whereas for the targeted sampling only one realization exists in this study. See Figure 3, Figures S2, S3 for graphical representations of the results and Table S1 for results of individual TCC events*.

a *For the combination of all realizations for all 11 TCC events in Figure 3 (in brackets without events 7 and 8)*.

b *For the 11 individual TCC events; see Table S1 for all values*.

For risk evaluation, it is important to not only detect periods of elevated bacterial concentrations, but also to quantify the peak concentration of a given event to judge the magnitude of pollution (Kistemann et al., 2002; Signor and Ashbolt, 2006). Therefore, we used the accuracy of estimating TCC peaks after precipitation as a second performance criterion for evaluating different monitoring strategies. In the following assessment, we evaluated the ratio R, i.e., the sampled maximum divided by the true maximum (Figure 2, Table 3, Figures S2, S3). As can be seen from Figure 3 and Table 3, the median peak estimation improved with increasing numbers of samples. For a bi-weekly sampling strategy, we found the median underestimation of the true peak concentration (i.e., 1–R) to be 36% for constant sampling intervals and 39% for random sampling during working hours (Table 3, Figure 3) with some variation between individual TCC events (Figures 2B,C, Figures S2, S3). Increasing the sampling frequency also increased the values for the 25 and 75% quartiles but strong underestimation was still observed in some realizations (Figure 3). This is due to the fact that the peaks in TCC were often sharp (in the range of hours) and thus even with a daily sampling strategy, the chances of not sampling close to the peak remained substantial. In tendency, sampling at constant intervals had a narrower range between the 25 and 75% quartiles compared to random sampling during working hours (Figure 3).

From the above analysis of different monitoring strategies applied to this particular spring, the following observations can be summarized:

1. Increasing the sampling frequency strongly increased the probability of detecting precipitation-induced TCC events, decreased the median underestimation of peak concentrations, and narrowed the range of this underestimation (Table 3, Figure 3, Figures S2, S3, Table S1).
2. A bi-weekly sampling strategy resulted in average detection probabilities >90% for TCC events and median underestimation of peak concentrations below 39%.
3. With a few specific exceptions, there was no substantial difference in performance between the strategies of constant sampling intervals (irrespective of working hours) and random grab samples during working hours for the same number of samples. This concurs with similar findings on chemical measurements in surface waters (Skeffington et al., 2015).

Although frequent sampling can achieve high detection probabilities and reliable peak estimations, large labor and cost requirements for these monitoring strategies renders them unrealistic for most practical applications. Hence, given limited resources and thus a limited number of samples that can be processed, sampling strategies must be optimized to focus on “meaningful” time periods. Furthermore, the goal of utilities and practitioners is not necessarily to detect every single contamination event, but to have the ability to detect any given event at any given time. In the following section, such a targeted monitoring strategy for precipitation-induced contamination events in karstic springs is considered.

### Optimizing contamination event detection through targeted sampling

The basic idea of targeted sampling is to trigger sample collection with data from an affordable and continuously available measurement of a relevant variable. For the specific example of karstic springs, precipitation or spring discharge measurements can be used as an early-warning signal (Figures 1A,D, 2A), indicating that a critical observation period is about to begin and thus (increased) sampling and analysis would be valuable (Madrid and Zayas, 2007; Stadler et al., 2008; Goldscheider et al., 2010). In the following analysis, we used precipitation events >10 mm in 24 h as the early warning criterion for triggering sampling. Subsequently a virtual sub-sampling of the online FCM data set (Figure 2A) was performed, with three samples collected at 24 h, 48 h, and 72 h after the event criterion was met.

With this approach, the probability to detect an individual contamination event was particularly high. In reality, short (< 24 h) TCC events, would be missed with this approach because they would be over before the first sample was collected (for example events 7 and 8 in Figure 2). It can be seen from Table 3 (with and without inclusion of events 7 and 8) that the targeted sampling strategy (*n* = 33) exceeded the probability of detecting the TCC events achieved with the two bi-weekly sampling strategies (*n* = 28) samples in the same observation period.

The median underestimation of the true peak concentration of a TCC event (i.e., 1–R) was 11% based on the highest TCC sample (Table 3; range for individual TCC events: 6–54%, Table S1). Thus, the targeted sampling performed 25%-points better than the bi-weekly constant interval sampling (Figure 3, Table 3). For individual TCC events, the peak estimations of the targeted sampling were 3–34%-points closer to the true values except for the minor events 7 and 8 (where the targeted sampling performed equally well and 9%-points worse respectively) (Table S1). In addition, the targeted sampling had much higher values and a narrower range for the 25 and 75% quartiles, which the other monitoring strategies would only reach with daily sampling (Figure 3, Table 3). In summary, the targeted sampling achieved a moderately higher detection probability of TCC events and a considerably better estimation of peak concentrations with a similar number of samples.

In order to capture every single TCC event in our data set, the targeted sampling strategy required 33 samples to be taken and analyzed (compared to 28 samples for a bi-weekly strategy). However, the strength of the targeted sampling lies in that it provides the utility with the choice to sample any given contamination event with high accuracy, rather than necessarily trying to detect every TCC event. Also, it is evident that if a system experiences fewer contamination events with longer periods in between events than seen in the above example, the targeted sampling will become considerably more efficient than the other two monitoring strategies.

### Considerations on generalization and system specific characteristics

The presented approach is considered to be generally valid for springs in geological settings and climatic regions that are frequently influenced by precipitation-induced contamination events (Stadler et al., 2008; Butscher et al., 2011; Delbart et al., 2014; Meus et al., 2014; Sinreich et al., 2014). However, the concepts discussed above are not limited to karstic springs, and can be developed for different systems (e.g., riverbank filtration, surface waters, treatment plants). In this regard, targeted sampling strategies always need to be adapted to the specific characteristics of the investigated system, and the following aspects should be considered:

1. The best *variable* to serve as the trigger for targeted sampling should be identified based on an assessment of existing data sets (e.g., precipitation data, operational data, online measurements of abiotic variables) and ideally also initial high-frequency microbial measurements (e.g., online flow cytometry or auto-sampler campaigns), if available.
2. The *threshold* of the trigger variable that leads to the start of sampling is crucial for the detection probability of events. Too low thresholds lead to unnecessary high numbers of samplings of baseline conditions whereas too high thresholds bare the risk of missing events. Initial high-frequency microbial measurements will support the identification of such thresholds.
3. The *lag time* between exceeding the trigger variable threshold and first targeted sampling should be selected such that the latter ideally always occurs well before the peak of the contamination event. Again, the system-inherent lag times should ideally be extracted from initial high-frequency microbial measurements (see also Delbart et al., 2014).
4. The *sampling interval* and the *number of samples* per event should be chosen such that the typical time scale of events in the investigated system are adequately covered. This means that the contamination peak always falls into the sampled period and thus depends on lag time, sampling interval and number of samples.

### Implications and practical recommendations

The suitability of TCC as a microbial process variable for improved understanding of water resources shown previously (Vital et al., 2012; Gillespie et al., 2014; Helmi et al., 2014) was extended to the investigation of short-term dynamics in the present study (Figure 1C). This highlights the value of measuring TCC (or similar cultivation-independent variables) automatically at high temporal resolution for microbial monitoring (Brognaux et al., 2013; Besmer et al., 2014; Besmer and Hammes, 2016). While TCC is not a direct hygienic indicator, it is one of the most direct microbial variables that can be measured online and is seen as a useful process variable to detect microbial dynamics. Using online microbial measurements to drive a targeted sampling approach allows the use of more advanced methods, e.g., for specific fecal indicator organisms or direct pathogen and/or community detection, at meaningful points in time and comparison to long-term records (Figures 1B,E; Stadler et al., 2008; Goldscheider et al., 2010; Butscher et al., 2011).

While permanent online monitoring offers considerable advantages (Janke et al., 2006) it will probably not be practically and financially feasible for microbial water quality monitoring in the near future – especially for smaller utilities. However, the two examples in our study clearly show that after initial high-frequency measurements during a limited period, future targeted monitoring can be based on a moderate number of samples, which can be handled with an auto-sampler or even manual grab sampling and conventional detection methods (e.g., indicator organisms). Our findings clearly support the growing awareness that conventional water quality monitoring approaches need to be improved to better support risk assessment and system optimization (Petterson and Ashbolt, 2016) and further confirm the high value of automated, targeted sampling to this end (Stadler et al., 2008).

Consequently, we propose the following practical recommendations for improved monitoring of microbial short-term dynamics in raw and treated drinking water systems:

1. Compile all available data and knowledge on possible dynamics in water quality (e.g., precipitation data; online measurements of discharge, conductivity; conventional monitoring records).
2. Prioritize systems or locations within a system (e.g., raw water sources, treatment plants) with assumed or known high variability in water quality based on the above data.
3. Perform monitoring at the highest possible temporal resolution for at least 10 events with available online tools for direct (e.g., TCC) or surrogate (e.g., turbidity, particle counter) detection of bacterial concentrations. In natural systems, such as karstic springs, the possible influence of seasonal differences should be taken into account when performing high-frequency monitoring campaigns.
4. From the high-frequency data set, establish the causes and the typical time scales of microbial dynamics.
5. Specifically, identify the most suitable early-warning variable (e.g., precipitation event, increase in spring discharge, increase in turbidity) as a trigger for targeted sampling.
6. Based on this compiled knowledge on the dynamics, test different alternative monitoring strategies on the high-frequency data set as was demonstrated in this study.
7. Implement the best alternative strategy that delivers sufficient information for the questions to be answered and is feasible with the resources available (see also: Ward et al., 1986; Harmel et al., 2006).

## Conclusions

- Bacterial concentrations in karstic spring water are usually low during dry weather periods but increase substantially after localized precipitation events.
- Conventional monitoring strategies, which are based on infrequent grab sampling, substantially underestimate both the number of contamination events and peak concentrations of bacteria during such contamination events.
- TCC is a useful measurement to track precipitation-induced contamination events in spring water.
- Emerging automated TCC measurement devices allow for the collection of high-frequency data sets over extended periods that can be used for a systematic evaluation of short-term dynamics and monitoring strategies.
- Optimization of monitoring strategies should be site specific and based on (1) systematic analysis of existing data sets and (2) pilot studies with the highest possible temporal and spatial resolution and information depth to enable an informed optimization of a targeted monitoring strategy.
- While higher sampling frequencies generally improve both the probability of event detection and the estimation of microbial peak concentrations, targeted sampling is most efficient and effective and can be applied flexibly for individual contamination events.

## Author contributions

Experimental design: MB, JS, and FH. Research: MB, JS, and FH. Data analysis: MB, FH, and CO. Writing/editing: MB, JS, FH, and CO.

### Conflict of interest statement

The authors declare that the research was conducted in the absence of any commercial or financial relationships that could be construed as a potential conflict of interest. The reviewer PS and handling Editor declared their shared affiliation.

## References

[B1] Astrom J. Petterson S. Bergstedt O. Pettersson T. J. R. Stenstrom T. A. (2007). Evaluation of the microbial risk reduction due to selective closure of the raw water intake before drinking water treatment. J. Water Health 5, 81–97. 10.2166/wh.2007.139 17890838

[B2] Bakker M. Vreeburg J. H. G. Palmen L. J. Sperber V. Bakker G. Rietveld L. C. (2013). Better water quality and higher energy efficiency by using model predictive flow control at water supply systems. J. Water Supply Res. Technol. Aqua 62, 1–13. 10.2166/aqua.2013.063

[B3] Besmer M. D. Hammes F. (2016). Short-term microbial dynamics in a drinking water plant treating groundwater with occasional high microbial loads. Water Res. 107, 11–18. 10.1016/j.watres.2016.10.041 27783929

[B4] Besmer M. D. Epting J. Page R. M. Sigrist J. A. Huggenberger P. Hammes F. (2016). Online flow cytometry reveals microbial dynamics influenced by concurrent natural and operational events in groundwater used for drinking water treatment. Sci. Rep. 6:38462. 10.1038/srep38462 27924920 PMC5141442

[B5] Besmer M. D. Weissbrodt D. G. Kratochvil B. E. Sigrist J. A. Weyland M. S. Hammes F. (2014). The feasibility of automated online flow cytometry for *in-situ* monitoring of microbial dynamics in aquatic ecosystems. Front. Microbiol. 5:265. 10.3389/fmicb.2014.00265 24917858 PMC4040452

[B6] Brognaux A. Han S. S. Sorensen S. J. Lebeau F. Thonart P. Delvigne F. (2013). A low-cost, multiplexable, automated flow cytometry procedure for the characterization of microbial stress dynamics in bioreactors. Microb. Cell Fact. 12:100. 10.1186/1475-2859-12-100 24176169 PMC4228430

[B7] Butscher C. Auckenthaler A. Scheidler S. Huggenberger P. (2011). Validation of a numerical indicator of microbial contamination for karst springs. Ground Water 49, 66–76. 10.1111/j.1745-6584.2010.00687.x 20180864

[B8] Casey D. Nemetz P. N. Uyeno D. H. (1983). Sampling frequency for water-quality monitoring-measures of effectiveness. Water Resour. Res. 19, 1107–1110. 10.1029/WR019i005p01107

[B9] Delbart C. Valdes D. Barbecot F. Tognelli A. Richon P. Couchoux L. (2014). Temporal variability of karst aquifer response time established by the sliding-windows cross-correlation method. J. Hydrol. 511, 580–588. 10.1016/j.jhydrol.2014.02.008

[B10] Do H. T. Lo S. L. Lan A. P. T. (2013). Calculating of river water quality sampling frequency by the analytic hierarchy process (AHP). Environ. Monit. Assess. 185, 909–916. 10.1007/s10661-012-2600-6 22437323

[B11] Farnleitner A. H. Wilhartitz I. Ryzinska G. Kirschner A. K. T. Stadler H. Mach R. L.. (2005). Bacterial dynamics in spring water of alpine karst aquifers indicates the presence of stable autochthonous microbial endokarst communities. Environ. Microbiol. 7, 1248–1259. 10.1111/j.1462-2920.2005.00810.x 16011762

[B12] Field M. S. Nash S. G. (1997). Risk assessment methodology for karst aquifers: (1) Estimating karst conduit-flow parameters. Environ. Monit. Assess. 47, 1–21.

[B13] Gauthier V. Barbeau B. Millette R. Block J.-C. Prévost M. (2001). Suspended particles in the drinking water of two distribution systems. Water Sci. Technol. 1, 237–245. Available online at: http://ws.iwaponline.com/content/1/4/237

[B14] Gillespie S. Lipphaus P. Green J. Parsons S. Weir P. Juskowiak K.. (2014). Assessing microbiological water quality in drinking water distribution systems with disinfectant residual using flow cytometry. Water Res. 65, 224–234. 10.1016/j.watres.2014.07.029 25123436

[B15] Goldscheider N. Pronk M. Zopfi J. (2010). New insights into the transport of sediments and microorganisms in karst groundwater by continuous monitoring of particle-size distribution. Geologia Croatica 63, 137–142. 10.4154/gc.2010.10

[B16] Harmel R. D. King K. W. Haggard B. E. Wren D. G. Sheridan J. M. (2006). Practical guidance for discharge and water quality data collection on small watersheds. Trans. Asabe 49, 937–948. 10.13031/2013.21745

[B17] Helmi K. Watt A. Jacob P. I. Ben-Hadj-Salah Henry A. Meheut G. Charni-Ben-Tabassi N. (2014). Monitoring of three drinking water treatment plants using flow cytometry. Water Sci. Technol. 14, 850–856. 10.2166/ws.2014.044

[B18] Hojris B. Christensen S. C. B. Albrechtsen H. J. Smith C. Dahlqvist M. (2016). A novel, optical, on-line bacteria sensor for monitoring drinking water quality. Sci. Rep. 6: 23935. 10.1038/srep23935 27040142 PMC4819223

[B19] ISO (2000). Detection and Enumeration of Escherichia Coli and Coliform Bacteria-Part 1: Membrane Filtration Method. Geneva: ISO 9308-1:2000.

[B20] ISO (2003). Horizontal Method for the Enumeration of Microorganisms-Colony-Count Technique at 30 Degrees. Geneva: ISO 4833:2003.

[B21] ISO (2005). General Requirements for the Competence of Testing and Calibration Laboratories. Geneva: ISO 17025:2005.

[B22] ISO (2006). Sampling-part 1: Guidance on the Design of Sampling Programmes and Sampling Techniques. Geneva: ISO 5667-1:2006.

[B23] ISO (2012). Conformity Assessment-Requirements for the Operation of Various Types of Bodies Performing Inspection. Geneva: ISO 17020:2012.

[B24] Janke R. Murray R. Uber J. Taxon T. (2006). Comparison of physical sampling and real-time monitoring strategies for designing a contamination warning system in a drinking water distribution system. J. Water Resour. Plan. Manage. 132, 310–313. 10.1061/(ASCE)0733-9496(2006)132:4(310)

[B25] Kistemann T. Classen T. Koch C. Dangendorf F. Fischeder R. Gebel J.. (2002). Microbial load of drinking water reservoir tributaries during extreme rainfall and runoff. Appl. Environ. Microbiol. 68, 2188–2197. 10.1128/AEM.68.5.2188-2197.2002 11976088 PMC127524

[B26] Liu Y. Zheng B. H. Wang M. Xu Y. X. Qin Y. W. (2014). Optimization of sampling frequency for routine river water quality monitoring. Sci China 57, 772–778. 10.1007/s11426-013-4968-8

[B27] Loftis J. C. Ward R. C. (1980). Water-quality monitoring-some practical sampling frequency considerations. Environ. Manage. 4, 521–526. 10.1007/BF01876889

[B28] Madrid Y. Zayas Z. P. (2007). Water sampling: traditional methods and new approaches in water sampling strategy. Trac Trends Analyt. Chem. 26, 293–299. 10.1016/j.trac.2007.01.002

[B29] Meus P. Moureaux P. Gailliez S. Flament J. Delloye F. Nix P. (2014). *In situ* monitoring of karst springs in Wallonia (southern Belgium). Environ. Earth Sci. 71, 533–541. 10.1007/s12665-013-2760-x

[B30] Naddeo V. Scannapieco D. Zarra T. Belgiorno V. (2013). River water quality assessment: implementation of non-parametric tests for sampling frequency optimization. Land Use Policy 30, 197–205. 10.1016/j.landusepol.2012.03.013

[B31] Page R. M. Besmer M. D. Epting J. Sigrist J. A. Hammes F. Huggenberger P. (2017). Online analysis: deeper insights into water quality dynamics in spring water. Sci. Total Environ. 599, 227–236. 10.1016/j.scitotenv.2017.04.204 28477479

[B32] Petterson S. R. Ashbolt N. J. (2016). QMRA and water safety management: review of application in drinking water systems. J. Water Health 14, 571–589. 10.2166/wh.2016.262 27441853

[B33] Prest E. I. Hammes F. Kotzsch S. van Loosdrecht M. C. M. Vrouwenvelder J. S. (2013). Monitoring microbiological changes in drinking water systems using a fast and reproducible flow cytometric method. Water Res. 47, 7131–7142. 10.1016/j.watres.2013.07.051 24183559

[B34] Pronk M. Goldscheider N. Zopfi J. (2006). Dynamics and interaction of organic carbon, turbidity and bacteria in a karst aquifer system. Hydrogeol. J. 14, 473–484. 10.1007/s10040-005-0454-5

[B35] Pronk M. Goldscheider N. Zopfi J. (2007). Particle-size distribution as indicator for fecal bacteria contamination of drinking water from karst springs. Environ. Sci. Technol. 41, 8400–8405. 10.1021/es071976f 18200870

[B36] R Development Core Team (2008). R: A Language and Environment for Statistical Computing. Vienna: R Foundation for Statistical Computin Available online at: http://cran.r-project.org/

[B37] Ryzinska-Paier G. Lendenfeld T. Correa K. Stadler P. Blaschke A. P. Farnleitner A. H.. (2014). A sensitive and robust method for automated on-line monitoring of enzymatic activities in water and water resources. Water Sci. Technol. 69, 1349–1358. 10.2166/wst.2014.032 24647204

[B38] Scheidleder A. (1999). Groundwater Quality and Quantity in Europe. Copenhagen: European Environment Agency.

[B39] Signor R. S. Ashbolt N. J. (2006). Pathogen monitoring offers questionable protection against drinking-water risks: a QMRA (Quantitative Microbial Risk Analysis) approach to assess management strategies. Water Sci. Technol. 54, 261–268. 10.2166/wst.2006.478 17037162

[B40] Sinreich M. Pronk M. Kozel R. (2014). Microbiological monitoring and classification of karst springs. Environ. Earth Sci. 71, 563–572. 10.1007/s12665-013-2508-7

[B41] Skeffington R. A. Halliday S. J. Wade A. J. Bowes M. J. Loewenthal M. (2015). Using high-frequency water quality data to assess sampling strategies for the EU Water Framework Directive. Hydrol. Earth Syst. Sci. 19, 2491–2504. 10.5194/hess-19-2491-2015

[B42] SLMB (2007). Method 1406.1: Detection of Enterococcus spp. (Schweizerisches Lebensmittelhandbuch). Berne: Federal Office for Public Health.

[B43] SLMB (2012). Method 333.1: Determining the Total Cell Count And Ratios Of High And Low Nucleic Acid Content Cells in Freshwater Using Flow Cytometry. (Schweizerisches Lebensmittelhandbuch). Berne: Federal Office for Public Health.

[B44] Stadler H. Skritek P. Sommer R. Mach R. L. Zerobin W. Farnleitner A. H. (2008). Microbiological monitoring and automated event sampling at karst springs using LEO-satellites. Water Sci. Technol. 58, 899–909. 10.2166/wst.2008.442 18776628 PMC3117179

[B45] Stevenson D. G. (1997). Water Treatment Unit Processes. London: Imperial College Press.

[B46] Storey M. V. van der Gaag B. Burns B. P. (2011). Advances in on-line drinking water quality monitoring and early warning systems. Water Res. 45, 741–747. 10.1016/j.watres.2010.08.049 20851446

[B47] Vital M. Dignum M. Magic-Knezeu A. Ross P. Rietueld L. Hammes F. (2012). Flow cytometry and adenosine tri-phosphate analysis: alternative possibilities to evaluate major bacteriological changes in drinking water treatment and distribution systems. Water Res. 46, 4665–4676. 10.1016/j.watres.2012.06.010 22763289

[B48] Vreeburg J. H. G. Schaap P. G. van Dijk J. C. (2004). Particles in the drinking water system: from source to discolouration, in 4th World Water Congress: Innovation in Drinking Water Treatment, Vol. 4 (Marrakesh; London), 431–438.

[B49] Ward R. C. Loftis J. C. Mcbride G. B. (1986). The data-rich but information-poor syndrome in water-quality monitoring. Environ. Manage. 10, 291–297.

