# Supplementary material for: Evaluating Monitoring Strategies to Detect Precipitation-Induced Microbial Contamination Events in Karstic Springs Used for Drinking Water
Source: Front Microbiol. 2017 Nov 22;8:2229. doi: 10.3389/fmicb.2017.02229 (PMC5703154; doi:10.3389/fmicb.2017.02229)
Supplement: Supplementary file 1 [file DataSheet1.PDF]

Supplementary information

Evaluating monitoring strategies to detect precipitation-induced microbiological contamination events in karstic springs used for drinking water

Michael D. Besmer, Frederik Hammes, Jürg A. Sigrist, Christoph Ort

Contents:

1. Example of conventional and temporally resolved monitoring from a second spring
2. Detection probabilities and peak estimations for different number of samples and monitoring strategies per TCC event
3. R-scripts used for the systematic analysis of monitoring strategies

1. Example of conventional and temporally resolved monitoring from a second spring

As was mentioned in the main manuscript, the observations made in the first spring were confirmed for the second example (Figure S1). However, here the majority of grab samples for Enterococcus (76 %) and *E. coli* (84 %) were above zero cfu and thus considered to be detections of precipitation-induced events. Consequently, the estimated detection rates were much higher (76 % and 84 % respectively) than for the first example. However, this is probably an overestimation. Many of the measurements > 0 cfu 100 ml^{-1} were of moderate concentration (average: 20.9 ± 26.2 cfu 100 ml^{-1}, median: 8.5 cfu 100 ml^{-1} for Enterococcus; average: 16.5 ± 22.2 cfu 100 ml^{-1}, median: 8.5 cfu 100 ml^{-1} for *E. coli*) while auto-sampler samplings after a precipitation event revealed much higher values exceeding 300 cfu 100 ml^{-1} for Enterococcus and 400 cfu 100 ml^{-1} for *E. coli* (Figure S1E). This suggests that this spring experiences elevated concentrations of indicator bacteria even during dry-weather periods and does not indicate improved detection probabilities of quarterly grab sampling compared to the first spring. It further emphasizes the value of temporally resolved data sets to better interpret microbiological loads and specifically assess peak concentrations of bacteria and the temporal evolution of precipitation-induced contaminations.

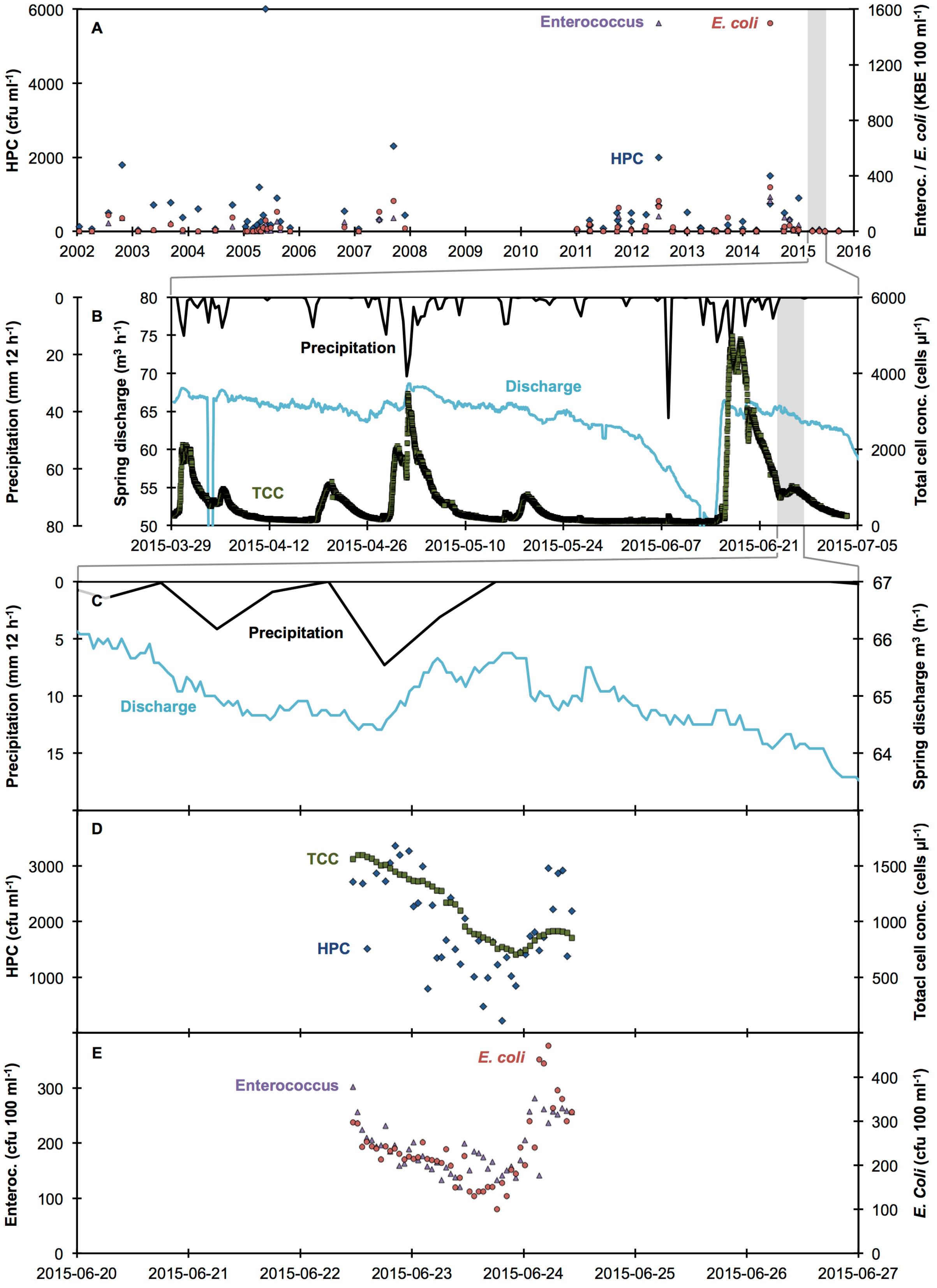

Figure S1: Analysis results of raw water from a spring with a record of high loads of indicator organisms: (**A**) conventional grab sampling (quarterly to monthly; 14 years; n = 76) analyzed with conventional plating methods for HPC (blue diamonds) and the indicator organisms Enterococcus (purple triangles), and E. coli (red circles), (**B**) online flow cytometry (green squares; n = 7'645), (**B**) & (**C**) precipitation and spring discharge measurements (hourly measurements; 12 months and a 10 weeks respectively), (**D**) & (**E**) auto-sampler measurements analyzed with conventional plating methods for HPC (blue diamonds) and the indicator organisms Enterococcus (purple triangles), and E. coli (red circles) as well as flow cytometry (green squares).

2. **Detection probabilities and peak estimations for different number of samples and monitoring strategies per TCC event**

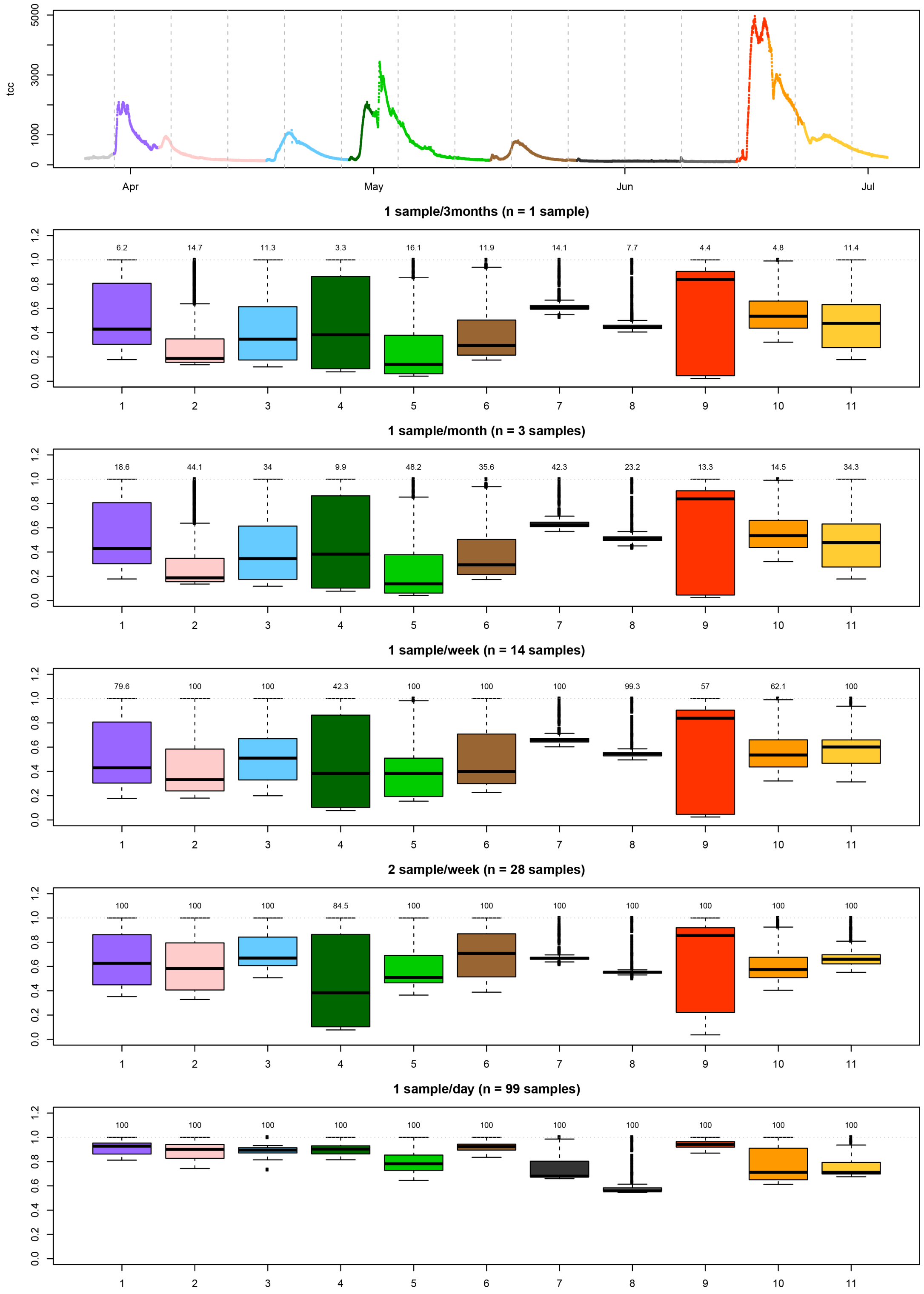

Figure S2: Probabilities of detecting TCC events and the distribution of the accuracy of peak concentrations of TCC (based on multiple realizations) for different sampling frequencies with the constant sampling interval. Numbers above boxes indicate the probability of TCC event detection (see **Error! Reference source not found.** and Table S1 for detailed results). Boxes represent 25 %, 50 % (i.e. median, black lines), and 75 % quartiles. Whiskers represent 1.5-fold interquartile ranges or minima / maxima when outside this range.

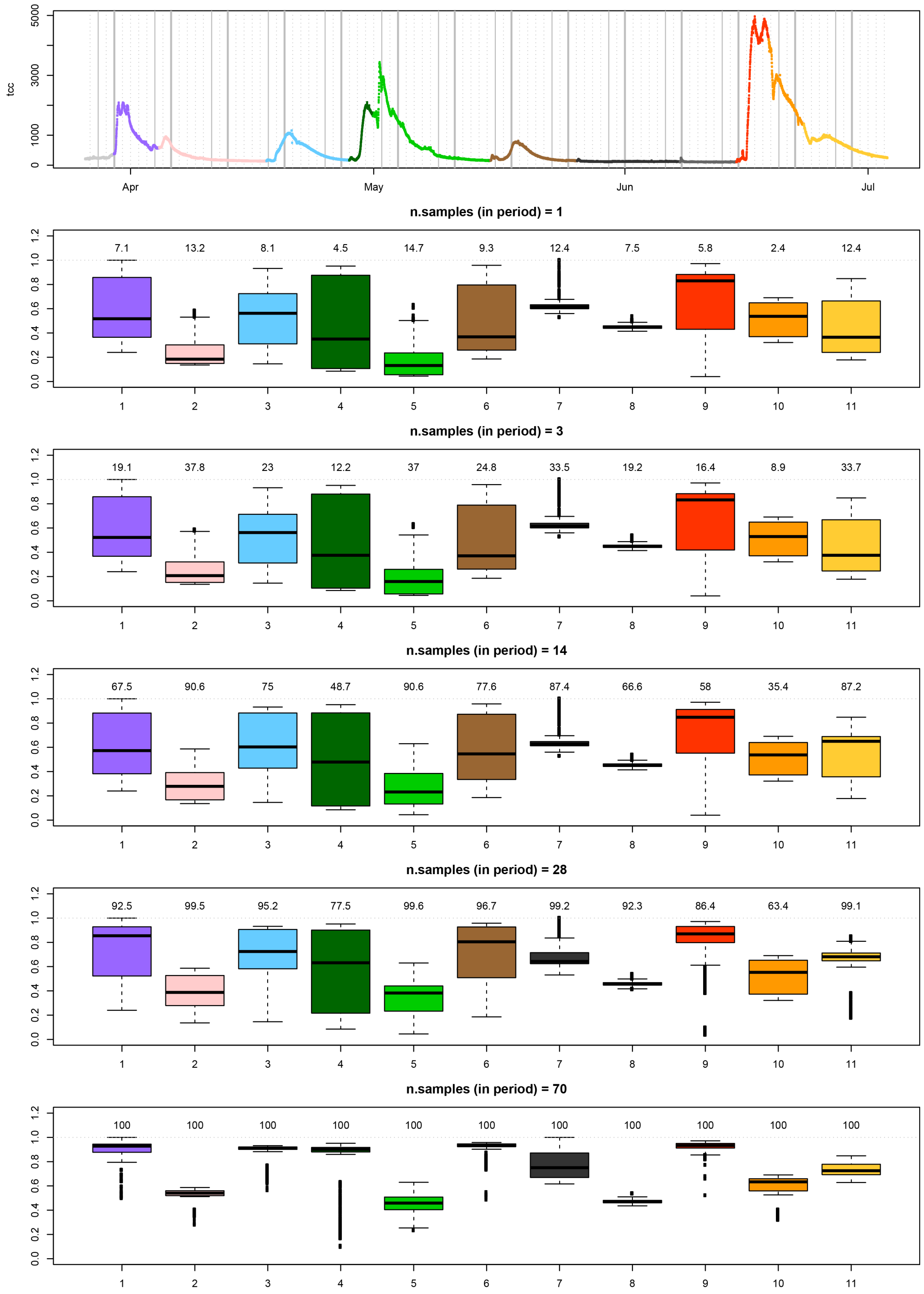

Figure S3: Probabilities of detecting TCC events and the distribution of the accuracy of peak concentrations of TCC (based on multiple realizations) for different sampling frequencies with random grab sampling during working hours. Numbers above boxes indicate the probability of TCC event detection (see **Error! Reference source not found.** and Table S1 for detailed results). Boxes represent 25 %, 50 % (i.e. median, black lines), and 75 % quartiles. Whiskers represent 1.5-fold interquartile ranges or minima / maxima when outside this range.

Table S1: Detailed results of accuracy of estimation of true peak concentrations (R) for the different evaluated monitoring strategies and TCC events. The three numbers for each sampling interval are 25 %, 50 % (i.e. median, bold), and 75 % quartiles in %. All three results are stated for targeted sampling since only one realization was possible. There, the measurements closest to the maximum are marked with bold numbers for each TCC event. Graphical representations can be found in **Error! Reference source not found.** Figure S2, and Figure S3 and summarized statistical values in **Error! Reference source not found.** and **Error! Reference source not found.**.

**Monitoring strategy**		**n**	**TCC event**										
			**1**	**2**	**3**	**4**	**5**	**6**	**7**	**8**	**9**	**10**	**11**
Constant interval			30	16	18	10	6	21	59	43	5	44	28
	Quarterly	1	**43**	**19**	**35**	**38**	**14**	**29**	**61**	**44**	**84**	**54**	**48**
			81	35	61	86	38	50	62	46	90	66	63
			30	16	18	10	6	21	61	50	5	44	28
	Monthly	3	**43**	**19**	**35**	**38**	**14**	**29**	**62**	**51**	**84**	**54**	**48**
			81	35	61	86	38	50	65	53	90	66	63
			30	24	33	10	19	30	64	53	5	44	47
	Weekly	14	**43**	**33**	**51**	**38**	**38**	**40**	**66**	**55**	**84**	**54**	**60**
			81	58	67	86	51	71	67	55	90	66	66
			45	41	61	10	47	52	66	55	23	51	62
	Bi-weekly	28	**63**	**58**	**67**	**38**	**51**	**71**	**67**	**55**	**86**	**58**	**66**
			86	79	84	86	69	87	67	56	92	68	70
			86	83	87	86	73	90	67	56	92	65	70
	Daily	99	**93**	**90**	**89**	**90**	**78**	**92**	**68**	**56**	**94**	**71**	**71**
			95	94	91	93	85	94	80	58	96	91	79
Randomly (during working hours)			37	15	31	10	6	25	60	44	40	37	24
	Quarterly	1	**52**	**20**	**46**	**29**	**16**	**37**	**61**	**45**	**83**	**40**	**37**
			86	32	68	87	24	78	63	46	88	63	66
			37	15	32	11	6	27	60	44	38	37	25
	Monthly	3	**52**	**21**	**58**	**37**	**16**	**37**	**62**	**45**	**83**	**40**	**38**
			86	32	72	88	26	80	64	46	88	63	67
			38	17	43	12	13	34	61	44	61	37	36
	Weekly	14	**57**	**28**	**60**	**49**	**23**	**55**	**63**	**45**	**85**	**54**	**65**
			88	39	88	89	38	90	65	46	91	64	69
			52	28	58	23	23	51	62	45	80	37	65
	Bi-weekly	28	**85**	**39**	**72**	**86**	**38**	**81**	**64**	**46**	**87**	**56**	**68**
			93	52	91	90	43	93	71	47	93	65	71
			88	52	90	88	41	92	67	46	91	56	69
	Daily	70	**93**	**54**	**91**	**90**	**46**	**93**	**75**	**47**	**93**	**63**	**73**
			94	56	92	92	51	95	88	48	95	66	78
			**89**	**92**	14	11	**72**	26	**67**	45	4	**67**	65
Targeted		33	67	52	66	**93**	52	32	63	**46**	**92**	54	63
			43	37	**90**	79	44	**94**	59	43	85	44	**70**

3. R-scripts used for the systematic analysis of monitoring strategies

```
# ==============================================
# Data evaluation for Paper Besmer et al.
# R-Script written by Christoph Ort, SWW, Eawag
# ==============================================


# ================================================================
# Read in data files, set system variables and general definitions
# ================================================================

setwd("//eaw-homedirs/ortchris$/My Documents/Tenure_2011/Projects/michael_besmer_sampling")

load("autosampler_results.RData")
load("random_grabs_results.RData")


all.data <- read.table(file="33616_0060_Auswertung_Sampling_Strategy_2016-04-27_MB_CO.csv", sep=";",
header=TRUE, colClasses=c("character", "numeric", "numeric", "numeric"))

dates<-all.data$Date_Time_UTC
dates <- as.POSIXct(strptime(unlist(dates), "%d.%m.%Y %H:%M:%S"), tz="GMT")
tcc <- all.data$TCC_interpoliert
events <- all.data$Event
discharge <- all.data$Discharge_.m3.h.
rain <- all.data$Niederschlag_.mm.30min.

Sys.setlocale("LC_TIME", "English")


# manually defined TCC events and color assignment
# ================================================

event.def <- as.numeric(levels(factor(events)))
n.event <- max(event.def)

event.cols.def <- c("#CCCCCC",
                "#9966FF",
                "#FFCCCC",
                "#66ccff",
                "#006600",
                "#00cc00",
                "#996633",
                "#333333",
                "#666666",
                "#ff3300",
                "#ff9900",
                "#ffcc33")

event.cols <- events
```

```
source("michael_sampling_functions_and_unused.R")


# =========================================================
# plot error distributions for autosampler fix (Fig SI6)
# =========================================================

# calculate true max of each event and sample for autosampler fix
# ================================================================

for(i in event.def)
{
  event.cols[which(events==i)]<-event.cols.def[i+1]
}

true.max.event <- det.prob.1d <- det.prob.2w <- det.prob.1w <- det.prob.1m <- det.prob.3m <-
rep(NA,1,n.event)

sampled.max.event <- rel.perform.1d <- rel.perform.1w <- rel.perform.2w <- rel.perform.1m <- rel.perform.3m
<- list()

for(i in 1:n.event)
{
  true.max.event[i] <- max(tcc[which(events==i)])
  sel.event <- which(events==i)

  sampled.max.event[[i]] <- sample.reduced(x=dates[sel.event], y=tcc[sel.event], z=24*60,
e=events[sel.event])
  rel.perform.1d[[i]] <- sampled.max.event[[i]]/true.max.event[i]
  event.length <- max(dates[sel.event])-min(dates[sel.event])
  ff=1
  if(units(event.length)=="hours")
  {
    ff <- 24
  }
  det.prob.1d[i] <- as.numeric(event.length)*24*60/(24*60)/ff*100

  sampled.max.event[[i]] <- sample.reduced(x=dates[sel.event], y=tcc[sel.event], z=3.5*24*60,
e=events[sel.event])
  rel.perform.2w[[i]] <- sampled.max.event[[i]]/true.max.event[i]
  event.length <- max(dates[sel.event])-min(dates[sel.event])
  ff=1
  if(units(event.length)=="hours")
  {
    ff <- 24
  }
  det.prob.2w[i] <- as.numeric(event.length)*24*60/(3.5*24*60)/ff*100

  sampled.max.event[[i]] <- sample.reduced(x=dates[sel.event], y=tcc[sel.event], z=7*24*60,
e=events[sel.event])
  rel.perform.1w[[i]] <- sampled.max.event[[i]]/true.max.event[i]
  event.length <- max(dates[sel.event])-min(dates[sel.event])
  ff=1
  if(units(event.length)=="hours")
```

```
  {
    ff <- 24
  }
  det.prob.1w[i] <- as.numeric(event.length)*24*60/(7*24*60)/ff*100

  sampled.max.event[[i]] <- sample.reduced(x=dates[sel.event], y=tcc[sel.event], z=30*24*60, e=events[sel.event])
  rel.perform.1m[[i]] <- sampled.max.event[[i]]/true.max.event[i]
  event.length <- max(dates[sel.event])-min(dates[sel.event])
  ff=1
  if(units(event.length)=="hours")
  {
    ff <- 24
  }
  det.prob.1m[i] <- as.numeric(event.length)*24*60/(30*24*60)/ff*100

  sampled.max.event[[i]] <- sample.reduced(x=dates[sel.event], y=tcc[sel.event], z=3*30*24*60, e=events[sel.event])
  rel.perform.3m[[i]] <- sampled.max.event[[i]]/true.max.event[i]
  event.length <- max(dates[sel.event])-min(dates[sel.event])
  ff=1
  if(units(event.length)=="hours")
  {
    ff <- 24
  }
  det.prob.3m[i] <- as.numeric(event.length)*24*60/(3*30*24*60)/ff*100


}


pdf(paste("Fig_SI6_results_auto_sampler_fix_new.pdf",sep=""), height=14,width=10)
par(mfrow=c(6,1), mar=c(2,4,3,2))

plot(dates, tcc, col=event.cols, pch=20, cex=0.5, main="autosampler (fix timing)")
# par(new=TRUE)
# plot(dates, discharge, pch=20, cex=0.3, ylim=c(50,70), yaxt="n")
# axis(side=4)
# abline(h=66)
# points(dates[discharge>66], discharge[discharge>66],col="red")
first.day.week <- dates[format(dates, "%w") == 1 & format(dates, "%H") == "00" & format(dates, "%M") == "00"]
abline(v=first.day.week, col="grey", lty="dashed")

boxplot(rel.perform.3m, col=event.cols.def[-1], ylim=c(0,1.2), main="1 sample/3months (n = 1 sample)")
abline(h=1,col="grey", lty="dotted")
det.prob.3m[det.prob.3m>100]=100
text(x=event.def[-1], y=rep(1.1,event.def[length(event.def)]), round(det.prob.3m,1), cex=0.8)

boxplot(rel.perform.1m, col=event.cols.def[-1], ylim=c(0,1.2), main="1 sample/month (n = 3 samples)")
abline(h=1,col="grey", lty="dotted")
det.prob.1m[det.prob.1m>100]=100
text(x=event.def[-1], y=rep(1.1,event.def[length(event.def)]), round(det.prob.1m,1), cex=0.8)
```

```
boxplot(rel.perform.1w, col=event.cols.def[-1], ylim=c(0,1.2), main="1 sample/week (n = 14 samples)")
abline(h=1,col="grey", lty="dotted")
det.prob.1w[det.prob.1w>100]=100
text(x=event.def[-1], y=rep(1.1,event.def[length(event.def)]), round(det.prob.1w,1), cex=0.8)

boxplot(rel.perform.2w, col=event.cols.def[-1], ylim=c(0,1.2), main="2 sample/week (n = 28 samples)")
abline(h=1,col="grey", lty="dotted")
det.prob.2w[det.prob.2w>100]=100
text(x=event.def[-1], y=rep(1.1,event.def[length(event.def)]), round(det.prob.2w,1), cex=0.8)

boxplot(rel.perform.1d, col=event.cols.def[-1], ylim=c(0,1.2), main="1 sample/day (n = 99 samples)")
abline(h=1,col="grey", lty="dotted")
det.prob.1d[det.prob.1d>100]=100
text(x=event.def[-1], y=rep(1.1,event.def[length(event.def)]), round(det.prob.1d,1), cex=0.8)

dev.off()

save(rel.perform.3m,
    rel.perform.1m,
    rel.perform.1w,
    rel.perform.2w,
    rel.perform.1d, file="autosampler_results.RData")


# numbers for table in MS (summary for fixed sampling)
# =====================================================

paper.table <- matrix(NA,nrow=2, ncol=11)
rel.perform.2w
for (i in 1:n.event)
{
  a = round(median(rel.perform.2w[[i]], na.rm=T),2)
  b = round(quantile(rel.perform.2w[[i]], na.rm=T, 0.25),2)
  c = round(quantile(rel.perform.2w[[i]], na.rm=T, 0.75),2)
  paper.table[1,i] <- a
  paper.table[2,i] <- paste("(",b,"-",c,")",sep="")
}

write.table(paper.table, file="table_S1_autosampler_2w_median_25_75_quantile.csv", sep=";")



# ==========================================
# random grab sampling on workdays (Fig SI 7)
# ==========================================

n.real = 10000

n=1
random.grab.1 <- working.days.sampling(n)
n=3
random.grab.3 <- working.days.sampling(n)
n=14
random.grab.14 <- working.days.sampling(n)
```

```
n=28
random.grab.28 <- working.days.sampling(n)
n=70
random.grab.70 <- working.days.sampling(n)

save(random.grab.1,
    random.grab.3,
    random.grab.14,
    random.grab.28,
    random.grab.70, file="random_grabs_results.RData")

pdf(paste("Fig_SI7_results_working_days_grab_random_new.pdf",sep=""), height=14,width=10)
par(mfrow=c(6,1), mar=c(2,4,3,2))

plot(dates, tcc, col=event.cols, pch=20, cex=0.5, main="random grab samples during working hours")
# par(new=TRUE)
# plot(dates, discharge, pch=20, cex=0.3, ylim=c(50,70), yaxt="n")
# axis(side=4)
# abline(h=66)
# points(dates[discharge>66], discharge[discharge>66],col="red")
first.day.week <- dates[format(dates, "%w") == 1 & format(dates, "%H") == "00" & format(dates, "%M") ==
"00"]
abline(v=first.day.week, col="grey", lwd=2)
saturday <- dates[format(dates, "%w") == 6 & format(dates, "%H") == "00" & format(dates, "%M") == "00"]
abline(v=saturday, col="grey")
tuesday <- dates[format(dates, "%w") == 2 & format(dates, "%H") == "00" & format(dates, "%M") == "00"]
wednesday <- dates[format(dates, "%w") == 3 & format(dates, "%H") == "00" & format(dates, "%M") == "00"]
thursday <- dates[format(dates, "%w") == 4 & format(dates, "%H") == "00" & format(dates, "%M") == "00"]
friday <- dates[format(dates, "%w") == 5 & format(dates, "%H") == "00" & format(dates, "%M") == "00"]
sunday <- dates[format(dates, "%w") == 0 & format(dates, "%H") == "00" & format(dates, "%M") == "00"]
abline(v=c(tuesday,wednesday,thursday,friday,sunday), col="grey", lty="dotted")
points(dates, tcc, col=event.cols, pch=20, cex=0.5)


n=1
boxplot(random.grab.1[[1]], col=event.cols.def[-1], ylim=c(0,1.2), main=paste("n.samples (in period) = ",
n,sep=""))
text(x=1:n.event, y=rep(1.1,n.event), round(random.grab.1[[2]],1))
abline(h=1,col="grey", lty="dotted")

n=3
boxplot(random.grab.3[[1]], col=event.cols.def[-1], ylim=c(0,1.2), main=paste("n.samples (in period) = ",
n,sep=""))
text(x=1:n.event, y=rep(1.1,n.event), round(random.grab.3[[2]],1))
abline(h=1,col="grey", lty="dotted")

n=14
boxplot(random.grab.14[[1]], col=event.cols.def[-1], ylim=c(0,1.2), main=paste("n.samples (in period) = ",
n,sep=""))
abline(h=1,col="grey", lty="dotted")
text(x=1:n.event, y=rep(1.1,n.event), round(random.grab.14[[2]],1))

n=28
```

```
boxplot(random.grab.28[[1]], col=event.cols.def[-1], ylim=c(0,1.2), main=paste("n.samples (in period) = ", n, sep=""))
abline(h=1,col="grey", lty="dotted")
text(x=1:n.event, y=rep(1.1,n.event), round(random.grab.28[[2]],1))

n=70
boxplot(random.grab.70[[1]], col=event.cols.def[-1], ylim=c(0,1.2), main=paste("n.samples (in period) = ", n, sep=""))
abline(h=1,col="grey", lty="dotted")
text(x=1:n.event, y=rep(1.1,n.event), round(random.grab.70[[2]],1))

dev.off()


# numbers for table in MS (summary for random grab sampling on working days)
# ================================================================================

paper.table <- matrix(NA,nrow=2, ncol=11)
# rel.perform.2w
for (i in 1:n.event)
{
  a = round(median(random.grab.28[[1]][,i], na.rm=T),2)
  b = round(quantile(random.grab.28[[1]][,i], na.rm=T, 0.25),2)
  c = round(quantile(random.grab.28[[1]][,i], na.rm=T, 0.75),2)
  paper.table[1,i] <- a
  paper.table[2,i] <- paste("(",b,"-",c,")",sep="")
}

write.table(paper.table, file="table_S1_28_random_grab_median_25_72_quantile.csv", sep=";")



#
==========================================================================================
==
# Figure 2 MS - subset of calculations above (basis, pimped afterwards in Illustrator)
#
==========================================================================================
==

cairo_pdf(paste("Fig_2MS_B_C_D_v3.pdf",sep=""), height=7.5,width=10)
par(mfrow=c(3,1), mar=c(2,4,3,2))

boxplot(rel.perform.2w, col=event.cols.def[-1], ylim=c(0,1.2), main="2 samples per week (n = 28 samples)", yaxt="n")
axis(side=2, at=seq(0,1,0.1), labels=c("0","","","","","0.5","","","","","1.0"), las=1)
abline(h=1,col="grey", lty="dotted")
det.prob.1m[det.prob.2w>100]=100
text(x=event.def[-1], y=rep(1.1,event.def[length(event.def)]), paste("(", round(det.prob.2w,1), ")", sep=""), cex=1)

# points(ratio.boxplot, pch=23, cex=1, bg="white")
points(result.matrix[9:19], cex=1.5, pch=21, bg="white")
```

```
n=28
boxplot(random.grab.28[[1]], col=event.cols.def[-1], yaxt="n", ylim=c(0,1.2), main=paste("grab working day n.samples (in period) = ", n, sep=""))
axis(side=2, at=seq(0,1,0.1), labels=c("0","","","","","0.5","","","","","1.0"), las=1)
abline(h=1,col="grey", lty="dotted")
text(x=1:n.event, y=rep(1.1,n.event), paste("(", round(random.grab.28[[2]],0), ")", sep=""))

n=1
boxplot(random.grab.1[[1]], col=event.cols.def[-1], yaxt="n", ylim=c(0,1.2), main=paste("grab working day n.samples (in period) = ", n, sep=""))
axis(side=2, at=seq(0,1,0.1), labels=c("0","","","","","0.5","","","","","1.0"), las=1)
abline(h=1,col="grey", lty="dotted")
text(x=1:n.event, y=rep(1.1,n.event), paste("(", round(random.grab.1[[2]],0), ")", sep=""))

dev.off()



# ============================================================
# evaluate number of events for Rünenberg Meteoschweiz Station
# ============================================================

ruenenberg <- read.table("2016-08-09 Meteo Data Rünenberg.csv", sep=";", header=TRUE)


date.time.ruenen <- ruenenberg[,3]
date.time.ruenen <- as.POSIXct(strptime(unlist(date.time.ruenen), "%d.%m.%Y %H:%M"), tz="GMT")

rain.ruenen <- ruenenberg[,4]

rain.ruenen[which(rain.ruenen==32767)] <- 0
rain.criterion = 10
lag.criterion = 24
frequency.criterion = 24
duration.criterion = 48
allow_break_of = 24 # how many hours can the 24h moving average be below required sum of rain
allow_break_of = allow_break_of*6 # in 10-minute steps
cond_last_trigger = 2 # do not define a new event before x days (related to the fact that sampling lasts over x days -> duration.criterion)

require(zoo)
sum24h.rain10 = append(rep(NA,(6*24-1)),6*24*rollmean(rain.ruenen,6*24))
sel10 <- sum24h.rain10>rain.criterion

sum10 <- rep(NA,length(date.time.ruenen))
sum10[sel10]<-16
sum10[!sel10]<-0
sum10=append(rep(NA,2*(allow_break_of-1)+(6*24)-allow_break_of),allow_break_of*rollmean(sum10[(6*24):length(date.time.ruenen)],allow_break_of))
sum10[sum10>0]=16


trigger=which(diff(sum10)>0)
units_diff=units(diff(date.time.ruenen[trigger]))
```

```
if(units_diff=="days")
{
  trigger_cond=date.time.ruenen[trigger[append(TRUE,diff(date.time.ruenen[trigger])>cond_last_trigger)]]
}
if(units_diff=="hours")
{
  trigger_cond=date.time.ruenen[trigger[append(TRUE,diff(date.time.ruenen[trigger])>cond_last_trigger*24)]]
}

sum10[sum10==0]<-NA

start.points = list()

for (n in 1:(length(trigger_cond)))
{
  start.points[[n]] <- trigger_cond[n]+lag.criterion*60*60
  #   if(!is.na(start.points[[n]]))
  #   {
  #     sample.points[[n]] <- seq(from=start.points[[n]],
  #                               to=start.points[[n]]+duration.criterion*60*60,
  #                               by=frequency.criterion*60*60)
  #   }
}

write.table(trigger_cond, file="all_start_points_of_events.csv", sep=";")



number.events <- function(datum1,datum2)
{
  length(which(trigger_cond>as.POSIXlt(unlist(as.POSIXct(datum1)), origin="1970-01-01", tz="GMT") &
               trigger_cond<as.POSIXlt(unlist(as.POSIXct(datum2)), origin="1970-01-01", tz="GMT")))
}

datum1 = "2002-01-01 01:00:00"
datum2 = "2016-01-01 01:00:00"
number.events(datum1,datum2)

datum1 = "2013-01-01 01:00:00"
datum2 = "2016-01-01 01:00:00"
number.events(datum1,datum2)

datum1 = "2015-03-28 01:00:00"
datum2 = "2015-06-24 01:00:00"
number.events(datum1,datum2)


# =================================
# FIG 3 MS comparison of performance
# =================================

cairo_pdf(paste("Fig_3_v3R.pdf",sep=""), height=4,width=8)
par(mar=c(5,5,3,2))
```

```
boxplot(result.matrix[9:19],
        xaxt="n",
        col="black",
        medcol="white",
        ylim=c(0,1),
        xlim=c(0.7,6.1),
        boxwex=0.4,
        at=5,
        xaxt="n",
        yaxt="n",
        main="white=autosampler, grey=random grab samples during working hours")

abline(h=quantile(result.matrix[9:19], c(0.25,0.5,0.75)),lty="dotted")

targeted=boxplot(result.matrix[9:19],
        add=TRUE,
        xaxt="n",
        col="black",
        medcol="white",
        ylim=c(0,1),
        xlim=c(0.7,6.1),
        boxwex=0.4,
        at=5,
        xaxt="n",
        yaxt="n",
        main="")

axis(side=1, at=seq(1,6,1), labels=c("1","3","14","28","33","99/70"))


constant=boxplot(unlist(rel.perform.3m),
        unlist(rel.perform.1m),
        unlist(rel.perform.1w),
        unlist(rel.perform.2w),
        unlist(rel.perform.1d),
        add=TRUE,
        boxwex=0.2,
        at=c(0.85,1.85,2.85,3.85,5.85),
        xaxt="n",
        xlab="number of samples in 3-month period",
        ylab="max(R) [-] over all events
        for all realisations",
        col="white")


random=boxplot(as.numeric(unlist(random.grab.1[[1]])),
        as.numeric(unlist(random.grab.3[[1]])),
        as.numeric(unlist(random.grab.14[[1]])),
        as.numeric(unlist(random.grab.28[[1]])),
        as.numeric(unlist(random.grab.70[[1]])),
        add=TRUE,
        boxwex=0.2,
        at=c(1.15,2.15,3.15,4.15,6.15),
```

```
        xaxt="n",
        yaxt="n",
        col="grey")

dev.off()

quantile(result.matrix[c(9:14,17:19)],c(0.25,0.5,0.75))
quantile(result.matrix[c(9:19)],c(0.25,0.5,0.75))


# ==========================================
# Values for table 2 in MS and table S1 in SI
# ==========================================

summary.table <- function(object)
{
  o1=round(quantile(object,na.rm=T,c(0.5,0.25,0.75)),2)*100
  o2=round(quantile(object[,c(1:6,9:11)],na.rm=T,c(0.5,0.25,0.75)),2)*100
  o3=round(min(apply(object, 2, function(x) median(x, na.rm=T))),2)*100
  o4=round(max(apply(object[,c(1:6,7:11)], 2, function(x) median(x, na.rm=T))),2)*100
  o5=round(min(apply(object, 2, function(x) median(x, na.rm=T))),2)*100
  o6=round(max(apply(object[,c(1:6,9:11)], 2, function(x) median(x, na.rm=T))),2)*100
  o7=round(apply(object, 2, function(x) quantile(x, c(0.25,0.5,0.75), na.rm=T)),2)*100
  return(list(o1,o2,o3,o4,o5,o6,o7))
}

quarterly.random = summary.table(as.matrix(random.grab.1[[1]]))
monthly.random = summary.table(as.matrix(random.grab.3[[1]]))
weekly.random = summary.table(as.matrix(random.grab.14[[1]]))
biweekly.random = summary.table(as.matrix(random.grab.28[[1]]))
daily.random = summary.table(as.matrix(random.grab.70[[1]]))

quarterly.fix = summary.table(matrix(data=unlist(rel.perform.3m), byrow=FALSE, nrow=8640, ncol=11))
monthly.fix = summary.table(matrix(data=unlist(rel.perform.1m), byrow=FALSE, nrow=2880, ncol=11))
weekly.fix = summary.table(matrix(data=unlist(rel.perform.1w), byrow=FALSE, nrow=672, ncol=11))
biweekly.fix = summary.table(matrix(data=unlist(rel.perform.2w), byrow=FALSE, nrow=336, ncol=11))
daily.fix = summary.table(matrix(data=unlist(rel.perform.1d), byrow=FALSE, nrow=96, ncol=11))


line1 <- cbind(t(paste(quarterly.fix[[1]],
                   " (",quarterly.fix[[2]],") ", sep="")),
               paste(quarterly.fix[[3]], " - ", quarterly.fix[[4]],
                  " (",quarterly.fix[[5]]," - ",quarterly.fix[[6]],")",
                  sep=""))

line2 <- cbind(t(paste(monthly.fix[[1]],
                   " (",monthly.fix[[2]],")", sep="")),
               paste(monthly.fix[[3]], " - ", monthly.fix[[4]],
                  " (",monthly.fix[[5]]," - ",monthly.fix[[6]],")",
                  sep=""))

line3 <- cbind(t(paste(weekly.fix[[1]],
                   " (",weekly.fix[[2]],")", sep="")),
               paste(weekly.fix[[3]], " - ", weekly.fix[[4]],
```

```
                    " (",weekly.fix[[5]]," - ",weekly.fix[[6]],")",
                    sep=""))

line4 <- cbind(t(paste(biweekly.fix[[1]],
                     " (",biweekly.fix[[2]],")", sep="")),
               paste(biweekly.fix[[3]], " - ", biweekly.fix[[4]],
                    " (",biweekly.fix[[5]]," - ",biweekly.fix[[6]],")",
                    sep=""))

line5 <- cbind(t(paste(daily.fix[[1]],
                      " (",daily.fix[[2]],")", sep="")),
                paste(daily.fix[[3]], " - ", daily.fix[[4]],
                     " (",daily.fix[[5]]," - ",daily.fix[[6]],")",
                     sep=""))


line6 <- cbind(t(paste(quarterly.random[[1]],
                     " (",quarterly.random[[2]],") ", sep="")),
               paste(quarterly.random[[3]], " - ", quarterly.random[[4]],
                    " (",quarterly.random[[5]]," - ",quarterly.random[[6]],")",
                    sep=""))

line7 <- cbind(t(paste(monthly.random[[1]],
                     " (",monthly.random[[2]],")", sep="")),
                 paste(monthly.random[[3]], " - ", monthly.random[[4]],
                     " (",monthly.random[[5]]," - ",monthly.random[[6]],")",
                     sep=""))

line8 <- cbind(t(paste(weekly.random[[1]],
                     " (",weekly.random[[2]],")", sep="")),
               paste(weekly.random[[3]], " - ", weekly.random[[4]],
                    " (",weekly.random[[5]]," - ",weekly.random[[6]],")",
                    sep=""))

line9 <- cbind(t(paste(biweekly.random[[1]],
               " (",biweekly.random[[2]],")", sep="")),
               paste(biweekly.random[[3]], " - ", biweekly.random[[4]],
                    " (",biweekly.random[[5]]," - ",biweekly.random[[6]],")",
                    sep=""))
line10 <- cbind(t(paste(daily.random[[1]],
               " (",daily.random[[2]],")", sep="")),
               paste(daily.random[[3]], " - ", daily.random[[4]],
                    " (",daily.random[[5]]," - ",daily.random[[6]],")",
                    sep=""))

write.table(rbind(line1,line2,line3,line4,line5,line6,line7,line8,line9,line10), file="table2_MS.csv", sep=";")


write.table(rbind(quarterly.fix[[7]],
                  monthly.fix[[7]],
                  weekly.fix[[7]],
                  biweekly.fix[[7]],
                  daily.fix[[7]],
                  quarterly.random[[7]],
```

```
monthly.random[[7]],
weekly.random[[7]],
biweekly.random[[7]],
daily.random[[7]]), file="table_S1_SI.csv", sep=";")
```

# from here on it is a separate file "michael_sampling_functions_and_unused.R" which must be sourced before execution of the code above.

```
# ==========================================================
# function for random grab sampling on workdays (Fig SI 7)
# ==========================================================

working.days.sampling <- function(n)
{

  sel <- format(dates, "%w") >= 1 & format(dates, "%w") <= 5 & format(dates, "%H") >"06" & format(dates, "%H") <"16"

  sel.working.days.dates <- dates[sel]
  sel.working.days.tcc <- tcc[sel]
  sel.working.days.events <- events[sel]
  sel.working.days.discharge <- discharge[sel]

  data.sel <- data.frame(sel.working.days.dates, sel.working.days.tcc, sel.working.days.discharge, sel.working.days.events)

  true.max.event <- sampled.max <- rep(NA,1,n.event)

  for(i in 1:n.event)
  {
    true.max.event[i] <- max(tcc[which(events==i)])
  }


  all.working.days <- as.numeric(unique(format(sel.working.days.dates, "%j")))

  result.matrix <- matrix(NA,nrow=n.real,ncol=n.event)
  for (a in 1:n.real)
  {
    sample.taken <- rep(NA,n)
    sampled.working.days <- sample(all.working.days,n,replace=FALSE)
    for(i in 1:n)
    {
      sel.day.x <- which(as.numeric(format(sel.working.days.dates, "%j"))==sampled.working.days[i])
      sample.taken[i] <- sample(sel.day.x,1)
    }
    for(j in 1:n.event)
    {
      sel.max.event <- sample.taken[data.sel[sample.taken,]$sel.working.days.events==j]
      sampled.max[j] <- max(data.sel$sel.working.days.tcc[sel.max.event])
    }
    result.matrix[a,] <- sampled.max/true.max.event
    sampled.max <- rep(NA,1,n.event)
  }
  result.matrix[result.matrix==-Inf]=NA
  detect.prob <- apply(!is.na(result.matrix), 2, sum)/n.real*100
  return(list(result.matrix, detect.prob))
}
```

```
# ====================================
# function for fix  sampling  (Fig SI6)
# ====================================

sample.reduced <- function(x,y,z,e)
{
  n.realisations <- z/15
  max.sampled <- rep(NA,n.realisations)
  #   if(n.realisations>length(y))
  #   {
  #     n.realisations <- length(y)
  #     max.sampled <- rep(NA,length(y))
  #   }

  duration <- 365/4*24*4

  x.e <- seq(from=x[1], by=15*60, length.out=duration)
  y.e <- y
  e.e <- e
  length.y.e <- length(y)
  sel.0 <- which(tcc<quantile(tcc,0.2))
  to.replace <- c((length.y.e+1):duration)
  n.to.replace <-length(to.replace)
  y.e[to.replace] <- sample(x=tcc[sel.0], size=n.to.replace, replace=TRUE)
  e.e[to.replace] <- 0
  # y.e[to.replace] <- sample(x=0, size=n.to.replace, replace=TRUE)

  start.times <- x.e[1:n.realisations]   #[!is.na(x[1:n.realisations])]

  for(j in 1:n.realisations)
  {
    times.sampled <- seq(start.times[j], by=z*60, length.out=(duration/n.realisations))
    sel <- match(times.sampled,x.e)
    max.sampled[j] <- max(y.e[sel], na.rm=T)
    if(sum(e.e[sel],na.rm=TRUE)==0)
    {
      max.sampled[j]=NA
    }
  }
  return(max.sampled)
}

# function to plot one realisation of fixed sampling (not used in final version)
# ==================================================================

plot.one.realisation.auto.fix <- function(day1, day2)
{
  sample.example <- (format(dates, "%H") == "02" &
                     format(dates, "%w") == day1 &
                     format(dates, "%M") == "00" ) |
    (format(dates, "%H") == "14" &
       format(dates, "%w") == day2 &
       format(dates, "%M") == "00")
```

```
  points(dates[sample.example], tcc[sample.example], bg=NA, pch=23, cex=0.6)
  ratio = rep(NA,n.event)
  for (i in 1:n.event)
  {
    sel.f <- which(events[sample.example]==i)
    ratio[i] = max(tcc[sample.example][sel.f],na.rm=T)/true.max.event[i]
  }
  return(ratio)
}

# trigger after x mm in 24h as start of sampling
# ============================================

if(TRUE)
{
  for(i in event.def)
  {
    event.cols[which(events==i)]<-event.cols.def[i+1]
  }

  cond_last_trigger = 2 # how many days ago should the last trigger be
  allow_break_of = 24 # how many hours can the 24h moving average be below required sum of rain
  allow_break_of <- allow_break_of*4 # in 15-minute steps

  true.max.event <- rep(NA,1,n.event)

  for(i in 1:n.event)
  {
    true.max.event[i] <- max(tcc[which(events==i)])
  }

  data.sel <- data.frame(dates, tcc, discharge, events)

  rain.criterion <- c(10)
  lag.criterion <- c(24)
  frequency.criterion <- c(24)
  duration.criterion <- c(48)


cairo_pdf(paste("Fig_2MS_A_",allow_break_of/4,"h_trigger_",cond_last_trigger,"day_new_cairo_v3.pdf",sep
=""), height=4,width=10)

par(mfrow=c(length(rain.criterion)*length(lag.criterion)*length(frequency.criterion)*length(duration.criterion),1),
mar=c(2,4,3,4))

  result.matrix                                                                                    <-
matrix(NA,nrow=length(rain.criterion)*length(lag.criterion)*length(frequency.criterion)*length(duration.criterio
n),ncol=8+n.event)

  sample.points = list()

  p=1

  for (j in 1:(length(rain.criterion)))
```

```
 {
   require(zoo)
   rain.interpol <- approxfun(1:length(rain),rain)
   rain15= rain.interpol(1:length(rain))/2
   sum24h.rain15 = append(rep(NA,95),96*rollmean(rain15,96))

   sel10 <- sum24h.rain15>rain.criterion[j]
   sum10 <- rep(NA,length(tcc))
   sum10[sel10]<-16
   sum10[!sel10]<-0
   sum10=append(rep(NA,2*(allow_break_of-1)+96-
allow_break_of),allow_break_of*rollmean(sum10[96:9503],allow_break_of))
   sum10[sum10>0]=16


   trigger=which(diff(sum10)>0)
   units_diff=units(diff(dates[trigger]))
   if(units_diff=="days")
   {
     trigger_cond=dates[trigger[append(TRUE,diff(dates[trigger])>cond_last_trigger)]]
   }
   if(units_diff=="hours")
   {
     trigger_cond=dates[trigger[append(TRUE,diff(dates[trigger])>cond_last_trigger*24)]]
   }

   sum10[sum10==0]<-NA

   for(k in 1:(length(lag.criterion)))
   {
     start.points = list()

     for (l in 1:(length(frequency.criterion)))
     {
       for (m in 1:(length(duration.criterion)))
       {
         for (n in 1:(length(trigger_cond)))
         {
           start.points[[n]] <- trigger_cond[n]+lag.criterion[k]*60*60
           if(!is.na(start.points[[n]]))
           {
             sample.points[[n]] <- seq(from=start.points[[n]],
                                   to=start.points[[n]]+duration.criterion[m]*60*60,
                                   by=frequency.criterion[l]*60*60)
           }
         }

         write.table(trigger_cond, file=paste("table_S1_break",allow_break_of/4,
                                        "trigger",cond_last_trigger,
                                        "rain",rain.criterion[j],
                                        "lag",lag.criterion[k],
                                        "int",frequency.criterion[l],
                                        "dur",duration.criterion[m],
                                        "_new.csv",
```

```
                                sep=""), sep=";")

        sample.points <- as.POSIXlt(unlist(sample.points), origin="1970-01-01", tz="GMT")

        data.sel.rain <- data.sel[as.numeric(data.sel$dates) %in% as.numeric(sample.points),]


        sampled.max.rain <- rep(NA,n.event)

        for(o in 1:n.event)
        {
          sampled.max.rain[o] <- max(data.sel.rain$tcc[data.sel.rain$events==o])
        }

        table_S1_SI <- round(matrix(data.sel.rain$tcc, nrow=3,ncol=11,byrow=FALSE))
        write.table(table_S1_SI, file="table_S1_SI_targeted.csv", sep=";")

        result.matrix[p,c(9:(8+n.event))] <- round(sampled.max.rain/true.max.event,2)
        result.matrix[p,c(1)] <- length(data.sel.rain[,1])
        result.matrix[p,c(2)] <- rain.criterion[j]
        result.matrix[p,c(3)] <- lag.criterion[k]
        result.matrix[p,c(4)] <- frequency.criterion[l]
        result.matrix[p,c(5)] <- duration.criterion[m]
        result.matrix[result.matrix=="-Inf"]=NA
        result.matrix[p,c(6)] <- round(mean(result.matrix[p,c(9:(8+n.event))], na.rm=T),2)
        result.matrix[p,c(7)] <- round(result.matrix[p,c(6)]/result.matrix[p,c(1)],4)
        result.matrix[p,c(8)] <- length(which(!is.na(result.matrix[p,c(9:(8+n.event))])))

        plot(dates,
             rain15,
             type="l",
             ylim=rev(range(c(0,10))),
             yaxt="n",
             xlab="",
             xaxt="n",
             ylab="",
             col="blue",
             yaxs="i",
             xaxs="i",
             las=1)

        axis(side=4, las=1, col="blue")

        first.day.month <- dates[format(dates, "%d") == "01" & format(dates, "%H") == "00" & format(dates,
"%M") == "00"]
        axis(side=1,at=first.day.month, lwd.ticks=2, labels=c("Apr", "May", "Jun", "Jul"))

        first.day.week <- dates[format(dates, "%w") == 1 & format(dates, "%H") == "00" & format(dates, "%M")
== "00"]
        axis(side=1,at=first.day.week, lwd.ticks=1.5, labels=FALSE)
        abline(v=first.day.week, lwd=0.5)

        start.day.week <- dates[format(dates, "%H") == "00" & format(dates, "%M") == "00"]
        axis(side=1,at=start.day.week, lwd.ticks=0.75, labels=FALSE)
```

```
        # abline(v=dates[trigger], col="grey")
        abline(v=trigger_cond,col="grey", lty="dashed", lwd=2.5)
        lines(dates,rain15,type="l",ylim=rev(c(0,10)),xaxt="n",yaxt="n",xlab="",ylab="", col="blue")
        mtext("rain [mm/15min]", side=4, line=3, col="blue")
        # points(dates, sum10, pch=20, cex=0.5)
        par(new=TRUE)
        #plot(dates, tcc, col=event.cols, pch=20, cex=0.5, main="")
        #length(trigger_cond)
        par(new=TRUE)

        plot(dates,
          tcc,
          col=event.cols,
          ylim=c(0,5500),
          ylab="tcc [-]",
          xaxt="n",
          yaxs="i",
          xaxs="i",
          bty="n",
          las=1,
          pch=19,
          cex=0.5,
          main=paste("rain[mm]=",
                  rain.criterion[j],
                  ", lag[h]=", lag.criterion[k],
                  ", int[h]=", frequency.criterion[l],
                  ", dur[h]=", duration.criterion[m],
                  ", n.sample=", length(data.sel.rain[,1]),
                  ", average.max", result.matrix[p,c(6)], sep=""))

        points(data.sel.rain$dates, data.sel.rain$tcc, pch=21, cex=1, bg="white")

        ratio.boxplot <- plot.one.realisation.auto.fix(day1="0", day2="3")

        p=p+1
      }
    }
  }
}

dev.off()

colnames(result.matrix) <- c("n.samples",
                    "rain",
                    "lag",
                    "int",
                    "dur",
                    "average.max",
                    "average.max_n.samples",
                    "n_events",
                    "event1",
                    "event2",
                    "event3",
```

```
                              "event4",
                              "event5",
                              "event6",
                              "event7",
                              "event8",
                              "event9",
                              "event10",
                              "event11")

  result.matrix

  sort.index=sort(result.matrix[,"average.max_n.samples"],index.return=T, decreasing=T)$ix
  result.matrix[sort.index,]

  write.table(result.matrix,
file=paste("results_rain_break_",allow_break_of/4,"h_trigger_",cond_last_trigger,"day_new.csv",sep=""),
sep=";")
}
```
